# A 140-year record of stream and valley sedimentation in the Whitewater River Valley, Minnesota, United States

**DOI:** 10.1038/s41597-026-07866-0

**Published:** 2026-07-17

**Authors:** Jimmy J. Wood, Andrew D. Wickert, Phillip H. Larson, Lawrence Svien

**Affiliations:** 1 Saint Anthony Falls Laboratory, University of Minnesota Twin Cities, 2 3rd Ave SE, Minneapolis, MN 55414 United States; 2 Department of Earth & Environmental Sciences, University of Minnesota Twin Cities, Suite 150, 116 Church St SE, Minneapolis, MN 55455 United States; 3 EARTH Systems Laboratory, Minnesota State University Mankato, 150 Carkoski Commons, Mankato, MN 56001 United States; 4 Natural Resources Conservation Service, United States Department of Agriculture, 3555 9th St NW, Rochester, MN 55901 United States; 5 Department of Geography, Pennsylvania State University, 302 Walker Building, University Park, PA 16802 United States

## Abstract

The introduction of European-style agriculture to the Upper Mississippi River Basin (U.S.) severely destabilized geomorphic systems and hydrologic regimes. Minnesota’s Whitewater River Valley exemplifies this phenomenon in microcosm as severe erosion, sedimentation, and flooding forced residents to abandon their villages and farmland within a century of habitation. Federal agencies studied this at the basin scale and, via transect boring and elevation surveys, built a near-140-year record of floodplain evolution along 107 cross sections. Here, we distill eight decades of historical primary source materials into digitized and geospatially registered (meta)data that capture the initial geomorphic state of the river-valley network and its response to anthropogenic forcing and landscape transformation between the 19th and 20th centuries. Patterns of aggradation and incision demonstrate floodplain construction and long-profile evolution—frequently simulated but rarely observed—alongside concurrent channel geometry adjustments. By converting this data into distributable and georeferenced digital forms, we provide an exemplary record of impacts to fluvial systems via agricultural intensification and ground-truth efforts to simulate and predict landscape evolution in accessible multi-format.

## Background & Summary

### Agriculturally accelerated surface processes

Anthropogenic activity drives geomorphic change by altering the natural regimes that influence Earth-surface processes. Although natural rates of erosion and deposition provide benefits (e.g., fertile soil production), accelerated surface-process rates can damage economic and ecologic systems^1^. Intensive land use related to agriculture has long been recognized to accelerate geomorphic processes^2–5^ through direct tillage and by promoting water runoff over subsurface infiltration^6^.

Worldwide, the 16th–19th century spread of European style agriculture via colonization yielded anomalously high rates of erosion and deposition^7^. In general, colonizers actively subverted well-established and lower-impact indigenous practices with geographically foreign ones characterized by a regime of land clearance, plowing, and grazing of significantly greater intensity^3^. Studies of this phenomenon across Africa^3^, the Americas^1,3^, Australia^3,7^, and New Zealand^3,8^ have shown that, despite the fact that indigenous, or pre-colonial, activity induced soil erosion, a general amplification of sediment fluxes^3^ and floodplain sedimentation—up to 33 times^3,4,8,9^—closely followed the arrival of European colonists^3^. The resultant deposits have been called post-settlement alluvium or, increasingly, legacy sediment (LS)^10,11^.

The land-use practices of European–Euro-American (hereafter, Euro-American) colonizers yielded vast quantities of LS across the contiguous United States (U.S.)^2^. Happ *et al*.^2^ and Dotterweich^3^ comprehensively, and collectively, reviewed the development of LS in the U.S. from the mid-18th century to the 1930s. The latter credits John Bartram—botanist–horticulturist–explorer—as the first to document the phenomenon in a mid-18th-century letter where he described how clearing the natural vegetative cover induced surface runoff and increased erosion^3,12^. Over the next two centuries, this phenomenon migrated west and became especially ubiquitous in the blufflands of the Upper Mississippi River Valley (UMRV) in the states of Minnesota, Wisconsin, Iowa, and Illinois^13^. The well-documented consequences of Euro-American land use in the Whitewater River Valley in southeastern Minnesota showcases the LS problems of the UMRV in microcosm.

### Erosion, deposition, & floods in the Whitewater River Valley

**Fig. 1:**
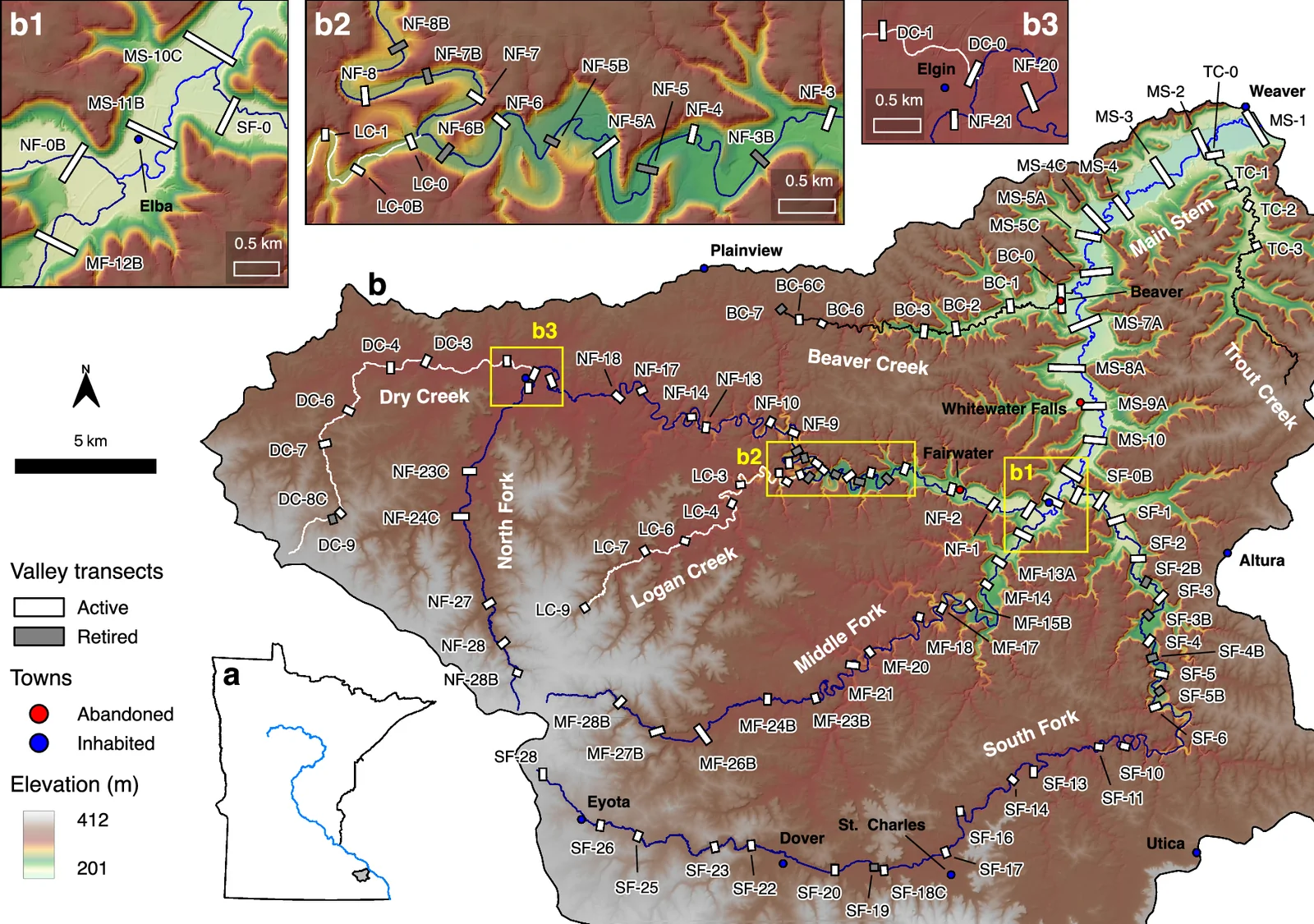
Geography of the Whitewater River Valley, Minnesota. Inset (**a**) shows the watershed’s location in southeast Minnesota (gray); the river drains approximately 830 km^2^ into the Mississippi River (blue line). The basin map (**b**), and insets **b1**–**b3**, display the 107 floodplain transects established throughout the Whitewater River network during the stream and valley sedimentation surveys of 1939 and 1965 conducted by the Soil Conservation Service and U.S. Geological Survey, respectively, under the leadership of Dr. Stafford Coleman Happ^65^. By 1994 (the last basin survey conducted by the SCS–Natural Resources Conservation Service), 94 transects were active (white) and the remaining 13 were retired from sampling (gray). Each transect on map (**b**) is labeled except where elements visually crowd (yellow rectangles) near the confluences of the three Forks of the Whitewater River near Elba (**b1**), the middle–lower valley of the North Fork and its confluence with Logan Creek (**b2**), and the confluence of the North Fork with Dry Creek, near Elgin (**b3**). Each river is labeled and distinguished by centerlines of contrasting shades of blue, white, and black. Follow any of the streams from their head to the Whitewater’s mouth and note the topographic change indicated by the shaded relief basemap; insets **b1**–**b3**, in reverse order, highlight this change from the relatively flat and open uplands of the upper valley, to the narrow and steep gorges of the middle valley, and finally to the wide and deep lower valley, respectively. Lastly, we show the basin’s towns—most, established in the mid-to-late-19th century—symbolized by their habitation status^74–76,85–91^. All abandoned towns are located in the lower valley of the Whitewater River, which was significantly affected by the erosion, sedimentation, and flood problems that plagued the basin throughout the late-19th through early-20th centuries^19–22^.

The river valleys of the Whitewater Watershed transition through three distinct physiographic regions (Fig. 1). The Whitewater River’s three forks—North, Middle, and South—and four tributaries—Dry, Logan, Beaver, and Trout Creeks—dominate the drainage network. The three forks coalesce to form the Main Stem Whitewater River near the town of Elba, and the four tributaries join the North Fork or Main Stem on their combined course to the Mississippi River. In general, these streams meander through upland plains, once prairie but now farm^14,15^, blanketed in Quaternary sediments^16^ and punctuated by outcrops of regional Cambrian–Ordovician sedimentary bedrock^17,18^. They dive through steep and narrow gorges, thickly wooded, walled by exposed bedrock^17,18^. As stream gradients lessen the gorges broaden into wide bluff-bordered bottomlands, once mixed prairie–forest^14^ but now covered in invasive monotypic grasses^19^ and the remnants of woods and farms. We call these three regions the upper, middle, and lower valleys, respectively.

With the indigenous Dakota forcibly displaced west (1851 Treaty of Traverse des Sioux)^20^ and the land mapped^14^, parcels went up for official sale on May 22, 1855^21^. By 1890, around 100 farms and five villages—Fairwater, Elba, Whitewater Falls, Beaver, and Weaver—were established in the valley^20^.

**Fig. 2:**
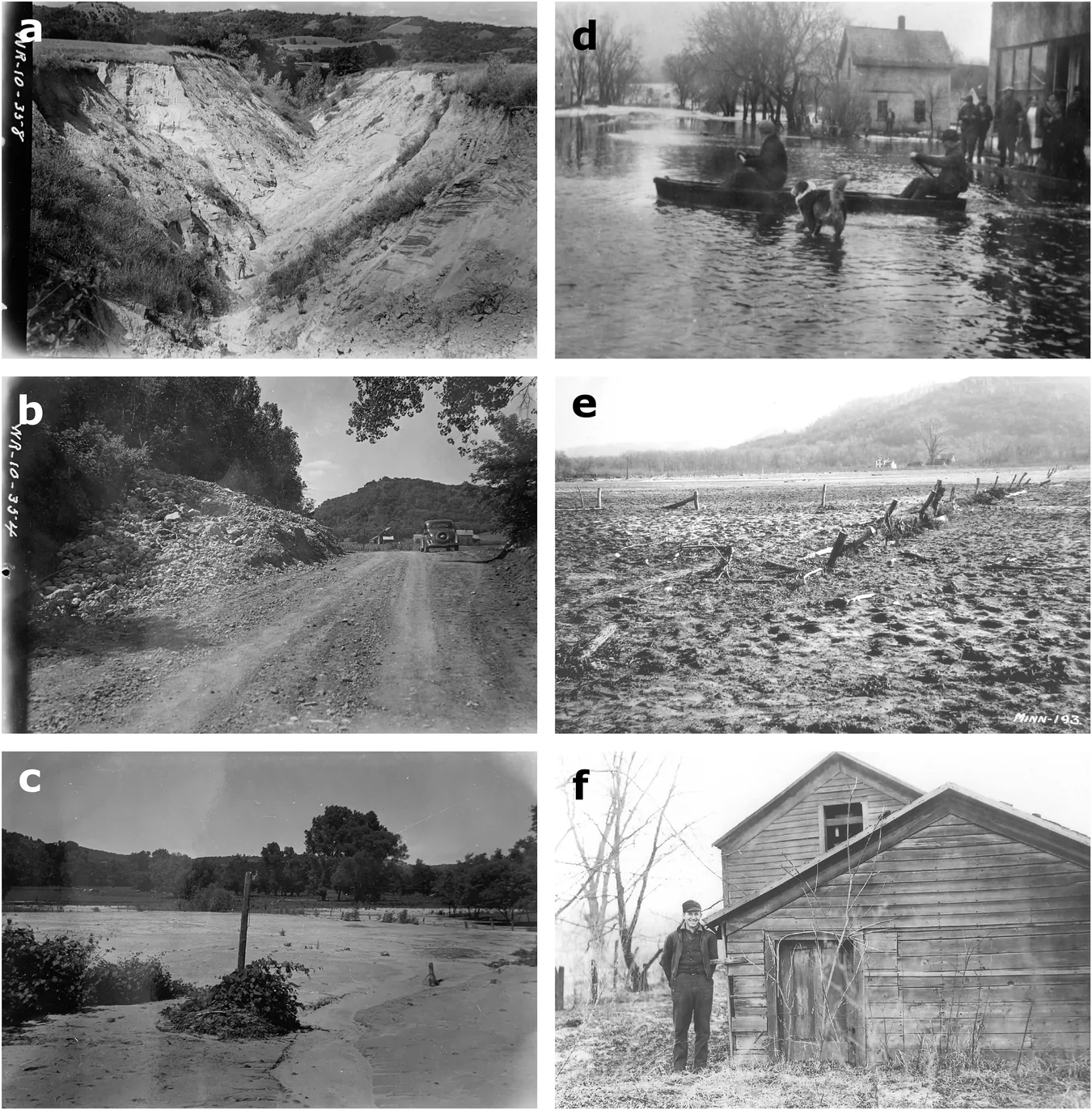
Agriculturally accelerated erosion, sedimentation, and flooding in the Whitewater River Valley, Minnesota (c. 1920s–1940s). (**a**) “View down the ‘big gully’ about 2 mi [3.2 km] SW of Weaver” (September, 1942)^92^. See the person standing in the center foreground for an indication of the magnitude of incision. (**b**) Boulder fan sourced from a bedrock gully developed across a highway west of Beaver (July 6, 1942). (**c**) View down the Young Cemetery sand fan, likely sourced from an upslope alluvial terrace, that blankets part of the Main Stem floodplain (July 6, 1942)^93^. (**d**) Residents of Beaver canoe through their inundated town (c. 1920s–1930s; photo courtesy of the Winona County History Center Laird Lucas Library & Archive)^94^. (**e**) Fence posts, likely on the Putnam farm NE of Beaver, damaged by floodwaters and buried in sediment (c. 1940s)—all of the visible floodplain is affected^22,95^. (**f**) An abandoned Beaver home partially buried by flood deposits (November 28, 1939)^22^. The individual standing on the flood deposits is significantly taller than the roof edge due to the deposition.

Following their immigration, Euro-American farmers cleared much of the natural vegetation for food production and initiated the valley’s geomorphic destabilization^19,21^. Within five decades of the initial sale year, gullies developed on the slopes and ridges of most farms^20^, as did alluvial fans downslope (Fig. 2a–c)^2^. By the 1930s–1940s, thousands of gullies required mitigation structures and hundreds of fans appeared in aerial photos. One fan was large enough to laterally displace 800 m of the Main Stem near Elba by 240 m^22^. High sediment loads from this widespread erosion overwhelmed fluvial sediment transport capacities, causing rapid aggradation that reduced the amount of water the rivers could carry within their banks. This reduced flow-conveyance capacity amplified flood heights and recurrence frequency (Fig. 2d)^2,22^. The town of Beaver flooded 28 times in 1938 alone^20^.

**Fig. 3:**
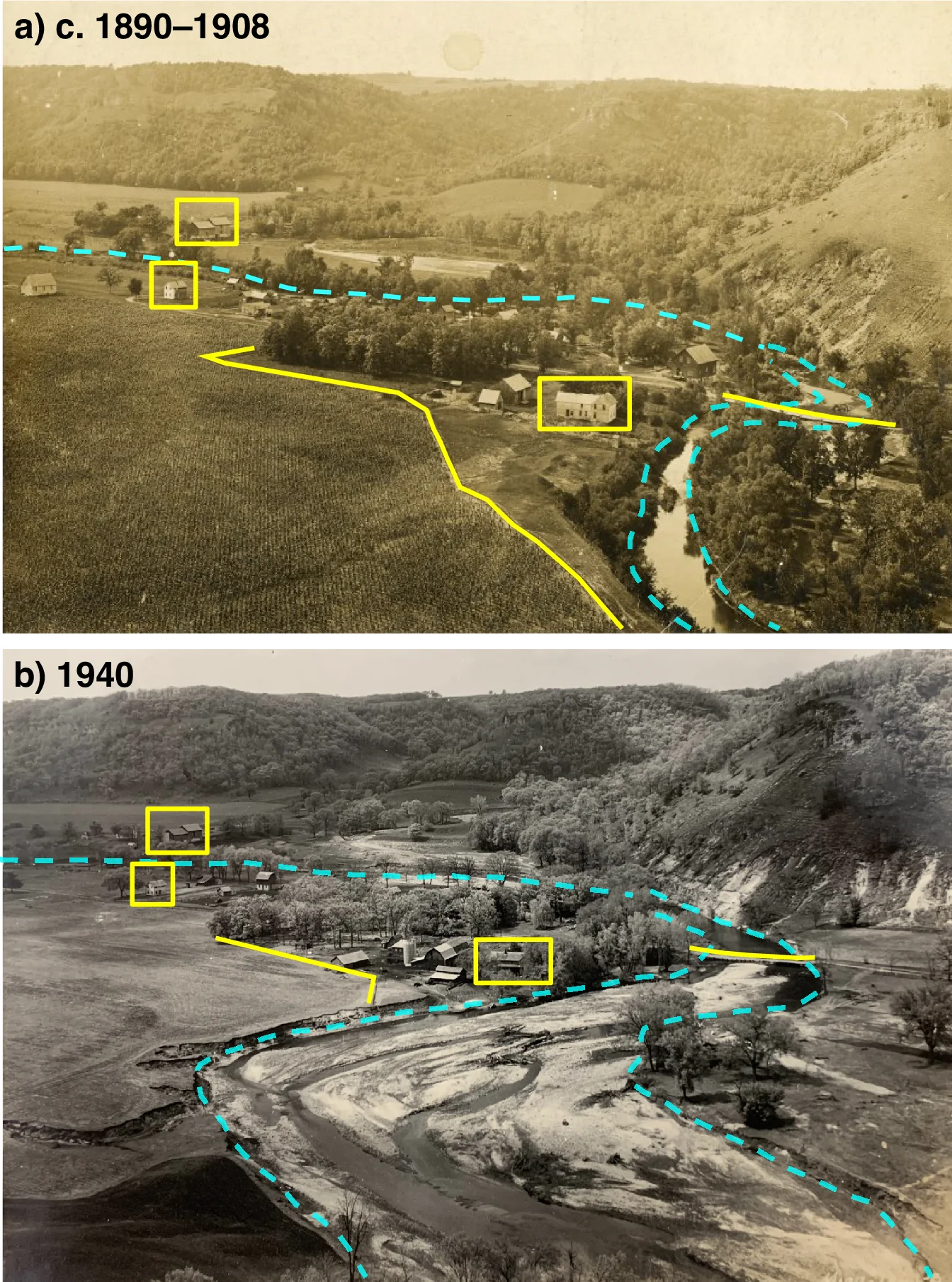
Geomorphic transformation of the North Fork Whitewater River at Fairwater, Minnesota, between c. 1890–1908 and 1940. (**a**) General condition of the valley and channel, c. 1890–1908, viewed from a rock ledge about 0.8 km SE of town (Photo courtesy of the Winona County History Center Laird Lucas Library & Archive)^96^. Note the degree of tree cover immediately adjacent to the channel, particularly in the foreground. Dashed cyan lines outline the approximate margins of the visible stream channel in the foreground and its centerline across the middle ground of each photo. Solid yellow annotations outline cultural features that are shared between the photos—the three rectangles highlight buildings, the right line indicates one of the North Fork’s bridges, and the left line shows the location of a farm fence. (**b**) Considerable change observed in 1940 from approximately the same point as photo **a**^97^. Bank erosion has widened the channel multiple times over and consumed much of the riparian zone noted in photo **a**. A significant portion of the farmstead at photo center has also been consumed. Note how its fence line is truncated by the widened channel.

Living with this environmental upheaval was fraught with financial difficulty. Erosion, sedimentation, and floods successively took land out of agricultural production as they carved it away or buried farms in sediment (Fig. 2e)^2,23^. Frequent inundation routinely damaged or destroyed infrastructure and private property as floodwaters washed away bridges and roads and filled establishments up to their second floors with alluvium (Fig. 2f)^22^. Figure 3 displays the severity of the damage experienced along the North Fork in Fairwater between c. 1890–1908 and 1940.

Many residents were compelled to abandon the valley and so sold their property back to the state^21^ with the aid of the Izaak Walton League^20^. This acquisition, completed by the 1960s^21^, yielded most of the considerable assemblage of public lands that exist in the Whitewater River Valley today^19^. Beaver, Fairwater, and Whitewater Falls ceased to exist. Weaver remains, though partially inundated by the formation of Pool 5 on the Mississippi River after the construction of Lock and Dam No. 5 in 1935^24^. Elba, originally built on a terrace about 3 m above the floodplain, now requires a levee for flood protection due to channel aggradation^22^.

### Federal interventions

Though the impacts of runaway soil erosion were recognized nationally and as worthy of federal intervention by 1908^25^, resources were not devoted to its mitigation for two more decades. First, the *Agricultural Department Appropriation Bill for 1930* appropriated funds to establish ten stations dedicated to soil conservation research^13,26^. One, the Upper Mississippi Valley Soil Conservation Experiment Station near La Crosse, Wisconsin, was established near the Whitewater basin^13^. Later government actions established the temporary Soil Erosion Service (SES) in 1933, to be responsible for conservation projects, and the permanent Soil Conservation Service (SCS) to supersede it in 1935^27^. These agencies also targeted the UMRV for study. The SES chose the Coon Creek watershed near La Crosse as the nation’s first basin-scale soil conservation demonstration project^28^, where newly developed conservation practices would be implemented on local farms^29^. Strip cropping^28^, conservation tillage, contour farming, crop rotation, and the construction of grassed waterways^20^ and terraces^28^, would later proliferate throughout the region^28^. Later, the SCS’s Sedimentation Division, intrigued by rumors of buried roads, bridges, and farms, extensively studied regional sedimentation conditions^22^.

Dr. Stafford Coleman Happ (1905–1992)—head of the Stream and Valley Section, Sedimentation Division, Office of Research, SCS—initiated a comprehensive basin-scale sedimentation survey of the Whitewater Watershed in the late 1930s^30^. In a 1939 memo, the *Whitewater Work Plan*, he outlined the project’s goals, which included the assessment of LS damage to farmland and its effect on flood conditions. The SCS then measured the magnitude and distribution of LS via auger boring and optical elevation surveys on around 100 floodplain transects along the Whitewater River and its principal tributaries to reach those goals^31^. Figure 1 displays all of the transects associated with this and the following studies.

Dr. Happ managed sixteen projects comparable in scope and design to the sedimentation survey of the Whitewater River Valley between 1935 and 1941. Of these, five were clustered in the UMRV^32^. Happ^32^ describes the collective relevance of this work through the declaration that, “Most of the pertinent information on valley sedimentation and related valley erosion is limited to the result of investigations by the Soil Conservation Service” from the timespan above. These years coincide with the sites’ first surveys and some small-scale resurveys (see Happ *et al*.^2^, McKelvey^29^, Adams^33–35^, Kunsman^36^, and Happ^30,37–39^). World War II’s global disruption shifted SCS priorities^40^ and precluded Dr. Happ’s ambitious plans for long-term monitoring through periodic resurveys^41^. In his 1996 memorial to Dr. Happ, Joseph B. Murphey (Agricultural Research Service [ARS] geologist) shared reflections by Drs. Luna B. Leopold and Stanley W. Trimble that both lament the post-war abandonment of these surveys and praise their significance in relation to our knowledge of sediment yield and fluvial geomorphology. Although Dr. Leopold encouraged Dr. Happ to preserve this survey data in the U.S. Geological Survey’s (USGS) Vigil Network database in the 1960s–1970s, data products for only three sites were added and with no original field data^42–44^.

In the 1960s, federal agencies such as the USGS and ARS started to revive the SCS’s stream and valley sedimentation studies—first, the Whitewater’s—with Dr. Happ again in charge^41^. Complete and partial resurveys in select basins continued until the mid-1990s, sometimes conducted by academic scientists and occasionally with Dr. Happ’s direct assistance or consultation (see Magilligan^6^, Svien^19^, Happ^32,45^, Trimble^46–48^, and Natural Resources Conservation Service [NRCS]^49^). Dr. Happ’s Whitewater River valley transects were surveyed in (1) 1939–1941, (2) 1964–1968, (3) 1975, (4) 1978, and (5) 1993–1995 for various projects.

The sedimentation data collected on Dr. Happ’s Whitewater Watershed transects exhibit significant spatiotemporal coverage. Survey techniques published by Happ *et al*.^2^ combined with the survey efforts listed previously have yielded a near 140-year record of geomorphic change that captures floodplain and channel dynamics in cross-section. The length of this interval not only provides a window into geomorphic adjustments on greater-than-human timescales, but also spans well-constrained and significant changes in local land use that enables the exploration of human impacts on surface-process rates. These land-use changes are (1) the introduction of Euro-American agriculture in the mid-1850s^21^, (2) the introduction of soil conservation practices in the UMRV in the mid-1930s^22^, and (3) the mass abandonment of the lower valley and its general conversion to public land by the 1960s^20^. The survey transects are also distributed across approximately 190 valley kilometers that span marked changes in land use, physiography, geology, and hydrology. This spatial coverage enables the investigation of erosion and deposition under diverse geographical conditions. Lastly, 21st-century data preservation efforts have yielded GNSS coordinates for the transects via monument markers, which has enhanced their field recovery and enabled geospatial analyses.

### Contemporary preservation

We present over eight decades of sedimentation survey data collected on 107 floodplain transects in the Whitewater River Valley of southeast Minnesota. We provide these data within conventional spreadsheets and GIS data files alongside the Python computer programs used to digitize and analyze them. The core dataset from the compiled surveys is largely divided into three components: (1) floodplain LS thicknesses from auger boring surveys, (2) hillslope–floodplain–channel elevation profiles from optical elevation surveys, and (3) monument coordinates from the GNSS survey. Each of these data components is available for each surveyed transect. We also provide metadata for the floodplain transects and their monuments. Lastly, we calculated mean-elevation-change rates per transect over various intervals between 1855 and 1994.

## Methods

Here we describe the 20th-century stream and valley sedimentation surveys of the Whitewater River Valley and 21st-century efforts to preserve the historical survey data and transect locations. The years-long surveys of 1939–1941, 1964–1968, and 1993–1995 will be denoted by 1939, 1965, and 1994, respectively. This is both convenient and highlights when most of the fieldwork for these efforts were completed. The 1975 and 1978 surveys, both isolated to transect MS-11B in Elba, were conducted by the SCS in response to high-profile floods and will not be described further. For the other field surveys, we focus on transect and monument establishment and/or recovery, auger boring surveys, and elevation surveys. For the data-preservation work, we highlight a monument GNSS survey conducted from 2008 to 2014, and historical data compilation and new data creation conducted from 2021 to 2025.

### 1939–1941: SCS

The SCS established 94 floodplain transects (or ranges) for their 1939 survey of the Whitewater River Valley. They were spaced about 1 mi (1.6 km) apart, on average, and located where LS existed in measurable quantities and was distinguishable from older deposits^19^. The ranges are aligned sub-orthogonally to the river-valley axis and extend between valley margins—hillslopes, terraces, etc.—to cover most to all of the floodplain^31^.

Transect IDs denote their location by identifying the stream they cross and their valley distance upstream of a reference point. An SCS memo dated November 16, 1940, describes the original naming convention; we have slightly modified it for consistency. The transects’ streams are indicated by an initialism of the stream name: MS (Main Stem), MF (Middle Fork), NF (North Fork), SF (South Fork), TC (Trout Creek), BC (Beaver Creek), LC (Logan Creek), and DC (Dry Creek). Valley distance to the nearest whole or quarter mile, likely measured by map scale, is indicated by a lone numeral (whole mile) or numeral-letter combination using A (+0.25 mile), B (+0.50 mile), or C (+0.75 mile), respectively. By this scheme, transect MS-4C is about 4.63–4.87 miles (4.75 miles) above the Main Stem’s mile-zero reference point. Main Stem and Middle Fork transect IDs are referenced to the U.S. Highway 61 bridge over the Whitewater River near Weaver. The rest of the streams’ mile-zero reference points are their downstream confluences with the Whitewater River.

To enable future recovery for long-term monitoring, transects were marked near both ends by survey monuments. A survey monument is a small structure, often emplaced in the earth, that houses a physically identifiable marker that may serve as a fixed positional reference^50^. The SCS monuments were almost exclusively stamped “1¼ [in] capped pipe[s] set in concrete” driven about 1.5 m into the subsurface. To aid monument (transect) recovery, surveyors established secondary markers on nearby trees and structures—nails, blazes, etc.—and recorded their positions relative to the pipes. Surveyors also measured transect bearings with Brunton compasses and annotated the lines onto aerial photos^41^. About 30% of the transects were monumented, exclusively in the lower valley and the lower-middle gorges of the Main Stem, the three Forks, and Beaver and Trout Creeks. Transects without monuments were blazed.

The monument naming convention identified the transect to which it belonged and the side of the transect it marked. The original scheme utilized the transect ID followed by the initial of a cardinal direction. Surveyors in 1965 added new directional components to supersede the former—L and R, presumably, for left and right—and even ones specific to replacement monuments—A, B, even LL. In the unified dataset presented here, we have standardized this scheme to reduce its complexity. The monuments’ transects are identified by the new scheme outlined above. The transect side marked by the monuments are exclusively labeled L or R for the left and right sides of the river from the perspective of an observer facing downstream. By this scheme, monuments 1-N and 1-S, the north and south side monuments for range 1 on the Main Stem, respectively, are now labeled MS-1-L and MS-1-R, respectively.

**Fig. 4:**
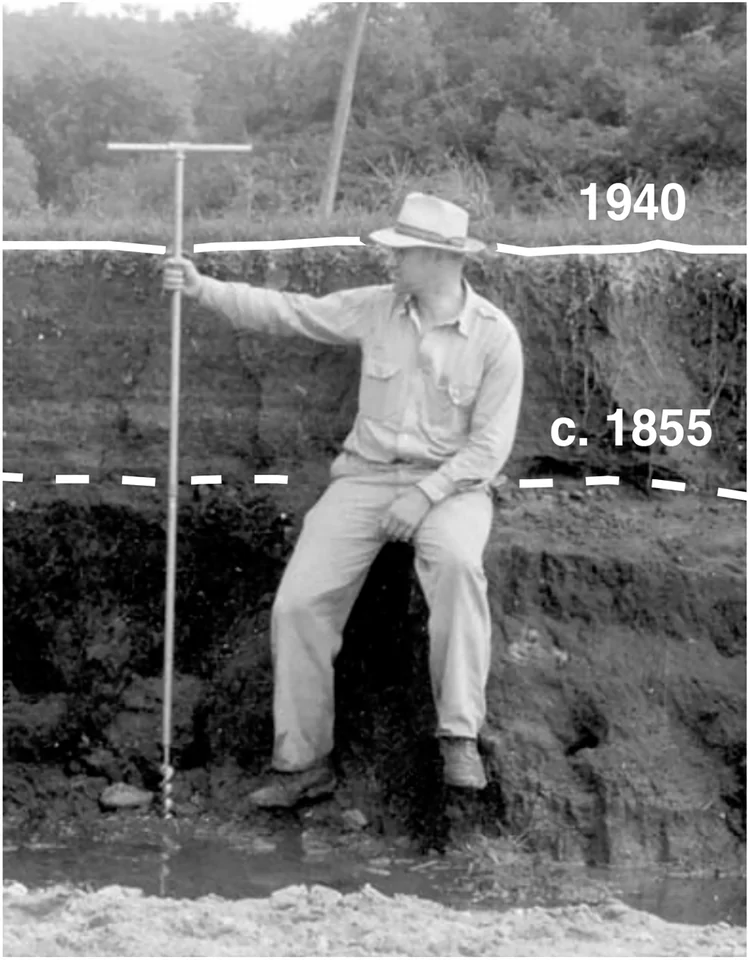
Legacy sediment (LS) stratigraphy in an unrecorded tributary of the Whitewater River near Beaver, Minnesota (August 29, 1940). Behind Dr. Stafford Coleman Happ, the cut bank exposes the contact between the old floodplain soil and recent LS (dashed line). By Happ *et al*.’s^2^ Principle 1, this is the pre-Euro-American floodplain surface (c. 1855). Between c. 1855 and 1940, about 60–90 cm of laminated LS buried this surface up to the elevation of the then-present floodplain (solid line; modified from Trimble^43^).

Along each transect, the SCS conducted an auger boring survey to measure LS thickness via methods published by Happ *et al*.^2^ First, they visually identified the subsurface base of LS accumulation by locating the contact between the younger LS, typically brown and laminated, and an older soil horizon, typically dark/black and relatively structureless (Principle one^2^; Fig. 4). The depth to this contact is assumed to equal the LS thickness.

**Fig. 5:**
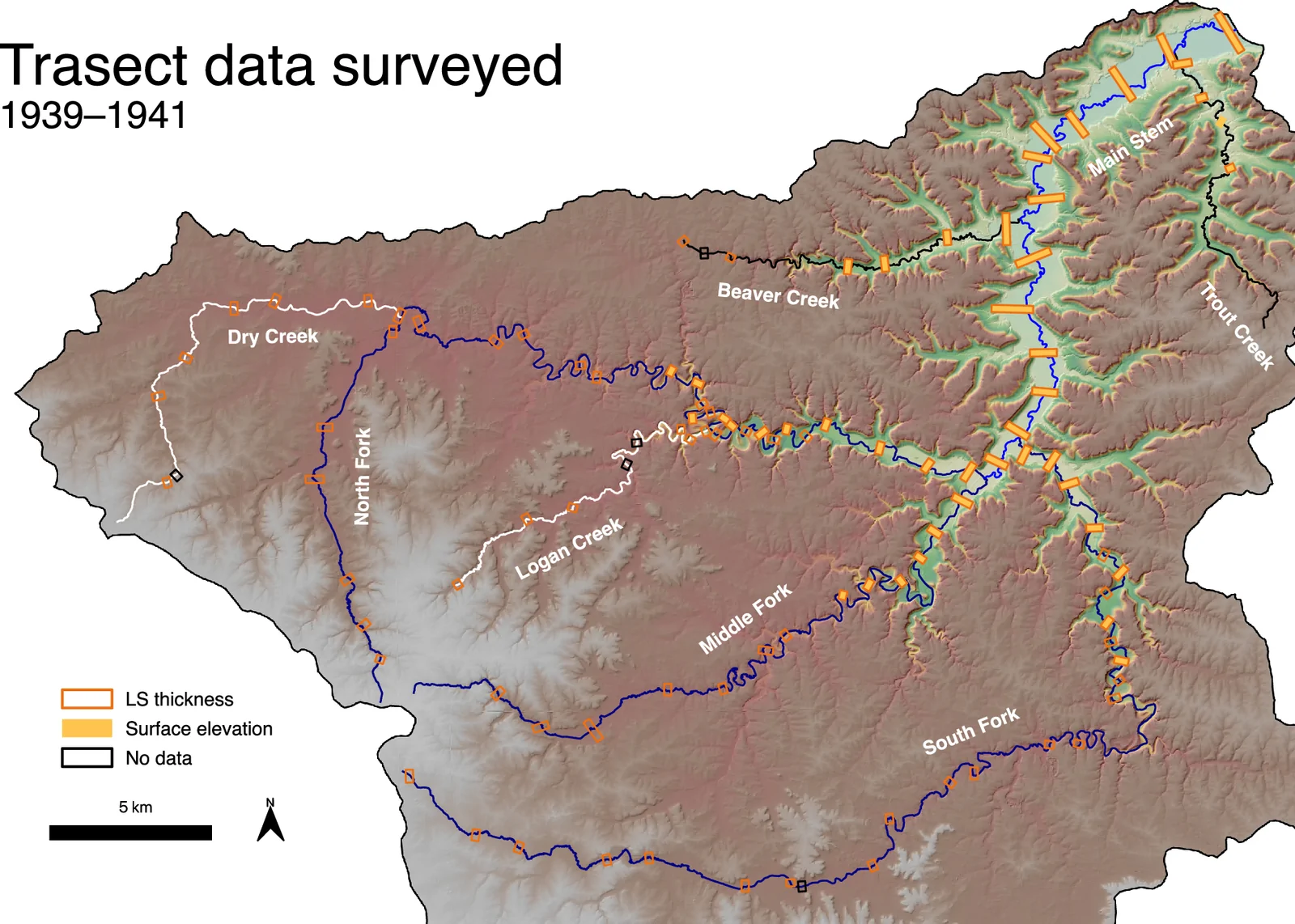
Transect data surveyed by the Soil Conservation Service during their stream and valley sedimentation survey of the Whitewater River Valley between 1939 and 1941—the first of three basin-wide surveys. Surveyors established 104 floodplain transects in the basin—94 for their study and ten for a related study conducted for the U.S. Army Corps of Engineers. They began surface-elevation surveys in the lower valley of the basin but were unable to extend them past the middle valleys and onto the upland surface. Their boring survey, to measure legacy sediment (LS) thickness, reached most of the transects. Five transects have “No data” for this survey: LC-3, -4; SF-18C; BC-6C; and DC-8C. We did not find boring logs for the two Logan Creek transects. The remaining three were not established until the U.S. Geological Survey’s survey of 1964–1968^65,86,88,91^.

The SCS bored transects with sheath/screw augers^31^ through a sampling scheme determined by local LS traits and range geometry (Fig. 5)^2^. Borehole spacing in the Whitewater Watershed typically varied between 8 and 30 m, but regularly extended both above this range—beyond 100 m—and below it to account for variations in LS thickness and the distinguishability of the target contact^2^. Transect length controlled the total number of boreholes augered but at least four per range were required^31^. When surveyors identified the “old soil” horizon, they recorded the LS depth, total borehole depth, and the borehole’s position down the transect (its station). Stations were measured by graduated tape, chaining, or pacing^32^. Though we assume that LS thicknesses were measured on all transects established during this survey, we did not find datasets for TC-2 or LC-3 and −4.

Optical elevation surveys, performed with spirit level and sighting scope, followed transect borings. These surveys were based on elevations established on transect markers, primarily the monument pipes but also nearby nails, which served secondary roles as elevation references, or benchmarks. In this case, “surveying” refers to the accurate measurement of locations, including elevations, on the Earth’s surface, but we will continue to use the term to refer to general field activities, for convenience^51^. To support their dual function, monuments were designed to be vertically stable and their pipes were designated Permanent Bench Marks (PBMs)^50^. Where available, PBM elevations were established from nearby existing benchmarks such as those established by the U.S. Coast and Geodetic Survey (USCGS) and USGS. Their elevations were mostly referenced to the National Geodetic Vertical Datum of 1929 (NGVD 29)—the then-current standard^52^—but some were referenced to the Fourth General Adjustment of 1912. Surveyors converted all elevations derived from the older datum to NGVD 29. Elsewhere, PBM elevations were left in a local, or assumed, datum relative to an arbitrary benchmark height, typically of 100 ft (30 m). Where a transect PBM was not yet established, a survey was referenced to nails in trees or another Temporary Bench Mark (TBM) near the range.

Surveyors measured a transect’s surface elevation at discrete points on the floodplain, stream channel, and lower parts of the valley walls to construct a continuous surface profile of the river valley. Elevations and sample stations were measured about every 6 m but also at visually apparent topographic breaks and at every previously dug borehole^31^. Where relevant, topography, hydrography, and/or infrastructure were also recorded at a station. In 1940, transect SF-1 was also extended on one side by about 187 m after it was surveyed the previous year. Surveyors performed bench level circuits to estimate measurement uncertainty per transect survey (the closure error) from the nearest USCGS, USGS, or other benchmark of reference^53^. Elevation surveys are limited to the transects of the lower valley and the lower-middle gorges but were conducted up valley beyond the extent of monument installation.

In the summer of 1940, the SCS established and sampled ten more transects, seemingly for a study conducted at the request of the U.S. Army Corps of Engineers (USACE). We gather that the USACE proposed the construction of two reservoirs on the North and South Forks, likely for flood control, that would inundate most of the valley length between transects NF-3 and -9, and SF-2 and -6, respectively. The SCS was asked to estimate future rates of sediment delivery to these proposed sites (Happ, S. C., personal communication, June 7 & 12, 1940). The new transects NF-3B, -5, -5B, -6B, -7B, -8B; and SF-2B, -3B, -4B, and -5B (see Fig. 1)—were established throughout the reservoir sites between the existing ranges; therefore, transect spacing in these sections is likely about 0.25–0.5 mi (0.4–0.8 km) rather than the typical 1 mi (1.6 km). The new transects were not monumented or elevation profiled but were augered for LS thickness. They were also never surveyed again, perhaps on account of having served their purpose.

### 1964–1968: USGS–ARS

**Fig. 6:**
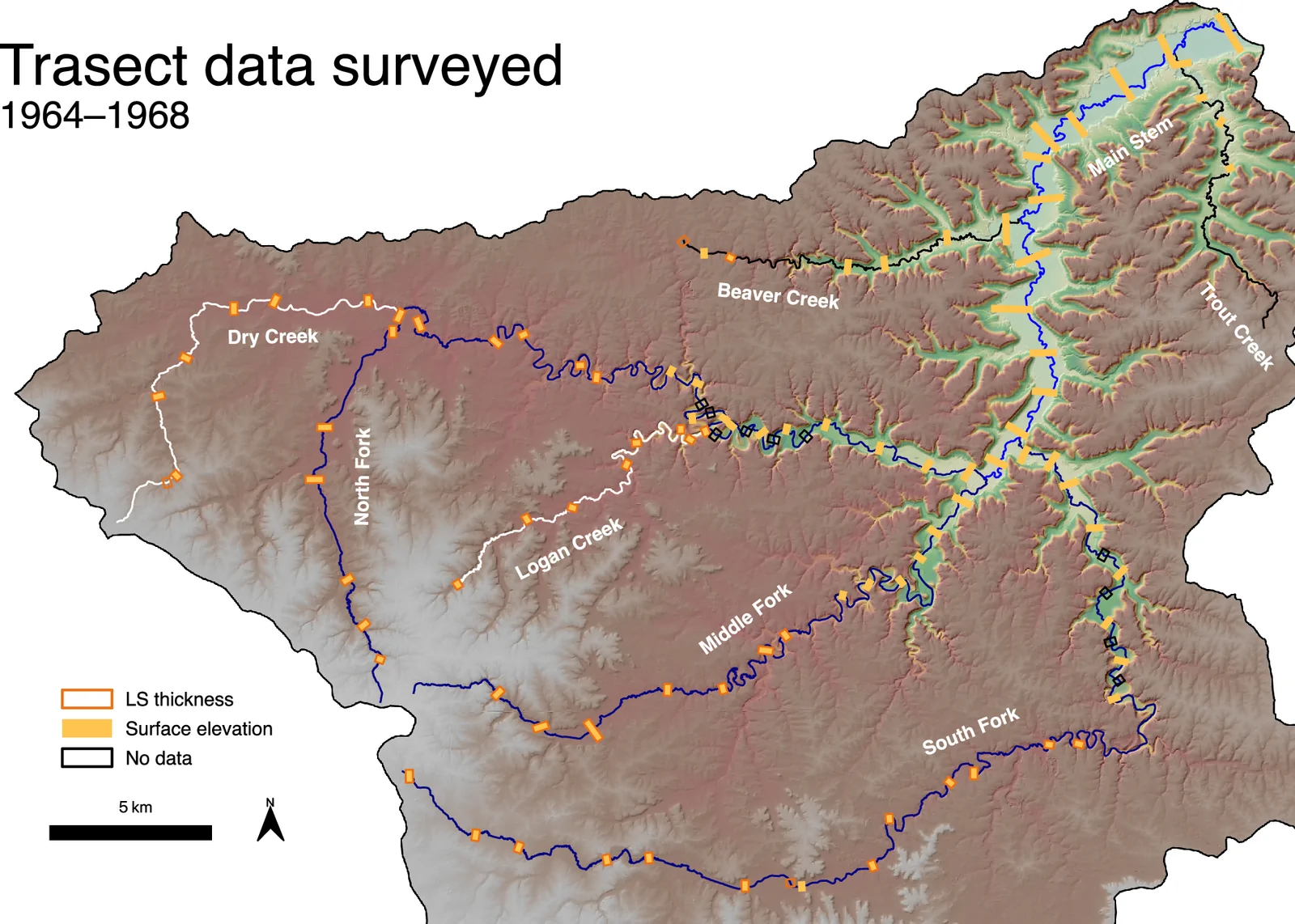
Transect data surveyed by the U.S. Geological Survey (USGS) during their stream and valley sedimentation survey of the Whitewater River Valley between 1964 and 1968 (below)—the second of three basin-wide surveys. Surveyors established three new floodplain transects and retired thirteen from sampling. Those retired include three transects from the original SCS study and all 10 from their related USACE project. This reorganization left 94 transects in the basin. The USGS surface elevation surveyed all 94 transects and re-bored the middle and upper valley lines that were not previously profiled in 1939 to, again, measure LS thickness. The ten transects that have “No data” for this survey are the retired North and South Fork ranges from the USACE study^65,86,88,91^.

Over two decades later, Dr. Happ, then employed by the USGS, initiated a Whitewater resurvey to support the agency’s interests to recover the SCS stream and valley sedimentation studies (Happ, S. C., personal communication, September 12, 1967). During this survey Dr. Happ transferred to the ARS. In late 1964, new elevation surveys were conducted on the lower and middle valley transects profiled in 1939 via the methods described previously (Fig. 6). Elevation survey closure errors were calculated almost exclusively from height comparisons on the SCS benchmarks rather than traditional bench level circuits. In the ideal situation, the surveyors compared a transect’s new survey-end benchmark elevation to that measured in 1939, the assumed standard—their difference yielded the closure error. Where only one monument was recovered, the comparison was made on the single, survey-start benchmark. The few original monuments that were not recovered were assumed to have been destroyed, such as by known highway improvements, and were replaced at other transect stations assumed to be more stable (Happ, S. C., personal communication, July 11, 1965). All monuments established during the USGS survey were of near-identical design to those from 1939, though they lacked the visible concrete casing that characterized the former.

From 1965 to 1968, data collection and monumentation were extended into the upper reaches of the Whitewater River network. The relevant transects were profiled, all in local datums, and monumented in 1965. Seventeen monuments on twelve transects were established laterally off range, typically at fence lines, anywhere from 0.3 to 44 m away from the transect. The twelve ranges are MF-20, -23B; NF-17, -20, -24C, -28, -28B; SF-16, -28; BC-6C; LC-7; and DC-8C. Though we lack definitive records, this likely occurred because of either local complications with monument installation, because landowners desired they be kept out of their way, or out of concern for benchmark stability (Happ, S. C., personal communication, July 28, 1971). Transect DC-1 is also the only survey line that was only monumented on one side. A second auger boring survey, to again measure LS thickness, was conducted between 1967 and 1968. Some transects also received additional elevation surveys. Range NF-24C was extended on both ends in 1968 by about 76–93 m. Range NF-28B was resurveyed and re-monumented in 1971 after nearby highway improvements buried its benchmarks and greatly altered the previously surveyed floodplain and channel surface (Brand, R. W., personal communication, July 12, 1971).

Thirteen transects were modified from their original 1939 configurations during the USGS survey. Transect NF-23C was laterally shifted by about 30 m to a location better suited to permanent monumentation. Ranges SF-10 and -23 were shifted about 30 m and 46 m on one end (rotated), respectively, to locations believed to be more representative of average erosion and sedimentation in their valley sections. Some of the other transects appear to have been relocated, seemingly due to difficulties encountered with range recovery such as blaze unintelligibility and topographic change over 25 years of tree growth and channel migration. Transects affected include MF-24B; NF-14; SF-11, -13; and DC-0, and -4. The 1965 traces of these transects were speculated to be within about 2–37 m from those established in 1939, some only on one end. No rationale seems to be recorded for the relocation of transects NF-20, DC-6, or LC-9, which were shifted about 210, 122, and 8 m (on one side), respectively. We speculate that LC-6 was also relocated, though by what distance and for what reason is not recorded. Lastly, NF-21 was shifted about 37 m, re-monumented, and resurveyed in NGVD 29 (originally local) in 1968 for roadway changes, similar to the circumstances that surround the 1971 resurvey of NF-28B.

The USGS also established three new transects—SF-18C, BC-6C, and DC-8C. Though the impetus is unclear, DC-8C is recorded as having replaced the nearby DC-9. Both were augered but only the new transect was monumented for future study and profiled. We assume that the same occurred with the two other new ranges—SF-18C replaced SF-19, and BC-6C replaced BC-7. These old lines were augered but the new ones were only monumented and profiled.

### 1993–1995: SCS–NRCS

In the early to mid-1990s, the SCS initiated another basin resurvey as a component of the interagency *Whitewater Watershed Project*. This endeavor was aimed towards the reduction of sediment and flood damages and the improvement of water quality in the Whitewater river network and Weaver Bottoms area of the Mississippi River. The SCS’s role was to manage a watershed improvement project under U.S. Public Law 83-566 (PL 83-566), the *Watershed Protection and Flood Prevention Act of 1954*^54,55^. Through the PL 83-566 project they would create a basin sediment budget with the USDA Agricultural Non-Point Source (AGNPS) pollution model^47,56^. Bill Thompson (Minnesota Pollution Control Agency project leader; Swanberg, S. L., personal communication, November 14, 1990) seems to have initiated the process to acquire some of the 1939 and 1965 survey materials with a letter to the long-retired Dr. Happ in 1990 (Happ, S. C., personal communication, August 24, [1990]). Over the next two years, Dr. Happ assembled records for mailing, while the SCS’s interest in the data grew (Nordstrom, G. R., personal communication, December 14, 1992). During a site field trip for project partners in 1991, Dr. Stanley W. Trimble presented his sediment-budget work for Coon Creek, Wisconsin^47^ (see Trimble^48,49,55,57,58^), and suggested that Dr. Happ’s Whitewater transects be resurveyed as a component of the new project (Bernard, J. M., & Bartels, R. M., personal communication, September 4, 1991). In 1993, the SCS formally requested the entire record of historical survey material from the ARS National Sedimentation Laboratory in Oxford, Mississippi (Murphey, J. B., personal communication, April 7 & October 22, 1993). The 1939, 1965, and new valley transect data they intended to collect would form a major component of their AGNPS model inputs^47^. The 1994 survey was the first to lack significant involvement from Dr. Happ due to his recent passing. During this resurvey, the SCS was renamed the NRCS.

**Fig. 7:**
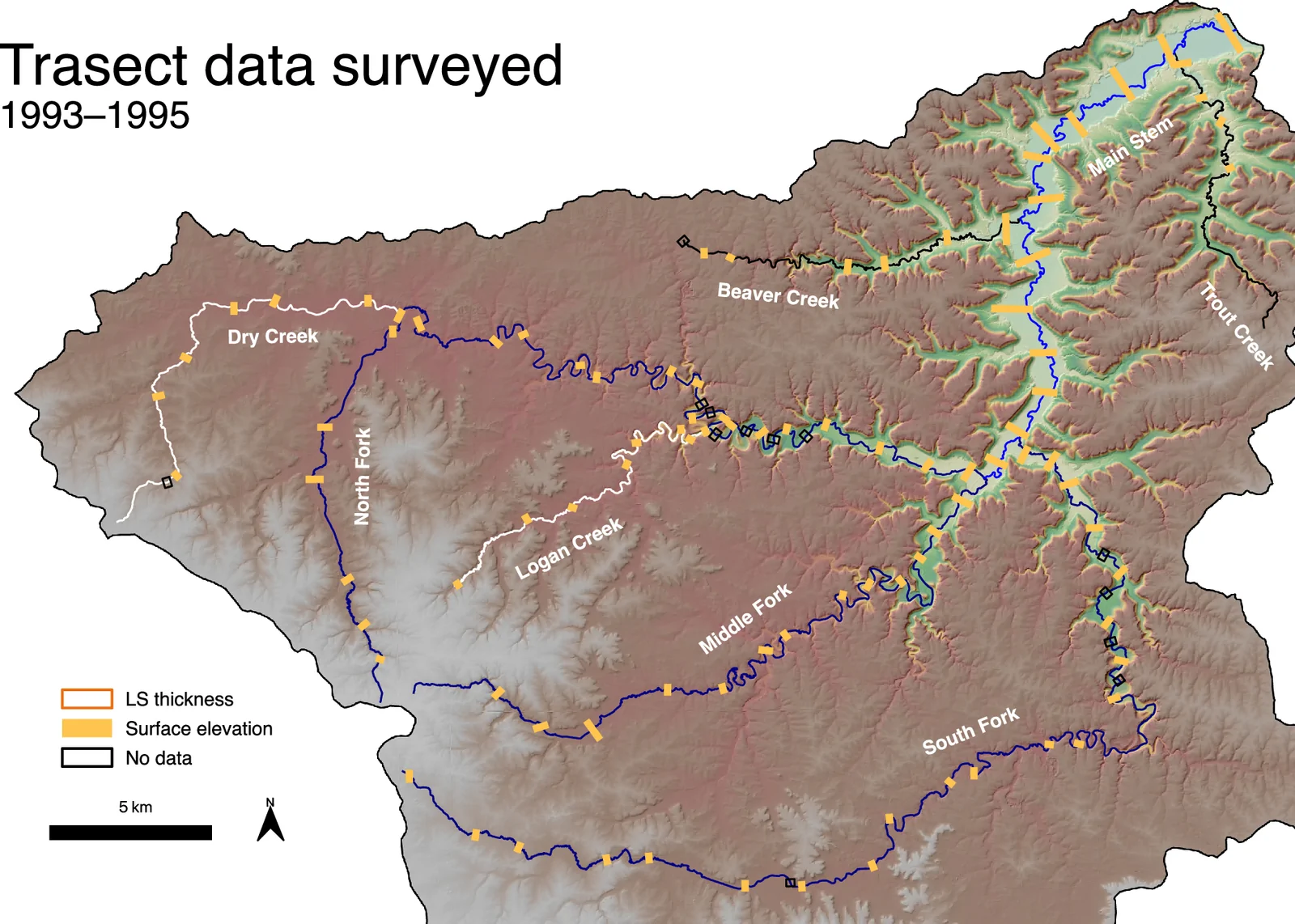
Transect data surveyed by the Soil Conservation Service–Natural Resources Conservation Service (SCS–NRCS) during their stream and valley sedimentation survey of the Whitewater River Valley between 1993 and 1995 (above)—the third and last basin-wide survey. The SCS–NRCS surface elevation surveyed all 94 transects—44 for the *Whitewater Watershed Project* sediment budget project and the remaining 50 to complete the dataset^46^. They did not bore any transects to measure LS thickness. The thirteen transects that have “No data” for this survey are the ten retired North and South Fork ranges from the USACE study, and the three ranges replaced and retired in 1965 on the South Fork, Beaver Creek, and Dry Creek^65,86,88,91^.

The SCS–NRCS (hereafter, NRCS) elevation surveyed all 94 valley transects (Fig. 7). Only 44 transects were actually surveyed for the PL 83-566 sediment budget^47^. The survey of the other 50 seems solely motivated by the interest of a few NRCS employees who worked on a volunteer basis on free weekends (Lorenzen, B., personal communication, June 6, 2001). Regardless of purpose, NRCS survey methods were largely identical to those employed previously, though they utilized modern electronic equipment (e.g., total station). They calculated closure errors via the method described for 1965 except where both transect benchmarks were unrecovered and not replaced. Eight transects were raised from local datums to NGVD 29: SF-4, -18C, -25; TC-0, -1, -2, -3; and DC-1.

Alongside their elevation surveys, the NRCS conducted limited remonumentation. Thirty monuments for 25 transects—mostly on the uplands—were not recovered, but only nine of those 30 were replaced. Instead of concrete encased pipes, the NRCS replaced monuments with rebar hammered into the earth. This much more casual approach to remonumentation, in number and design, makes economical sense—especially considering how most of the work was accomplished (i.e., volunteerism)—but has nevertheless diminished transect recovery potential and perhaps precluded the replacement monuments’ utility as benchmarks.

The NRCS also modified seven transects. Six were extended on one or both ends between about 61 and 238 m—MF-21, -24B, -26B; NF-18, -20; and SF-13. Part of transect DC-7 bounding the channel was partially shifted downstream about 8 m to avoid ground disturbance from a culvert crossing installed since 1965.

### 2008–2014: NRCS–Pennsylvania State University

**Fig. 8:**
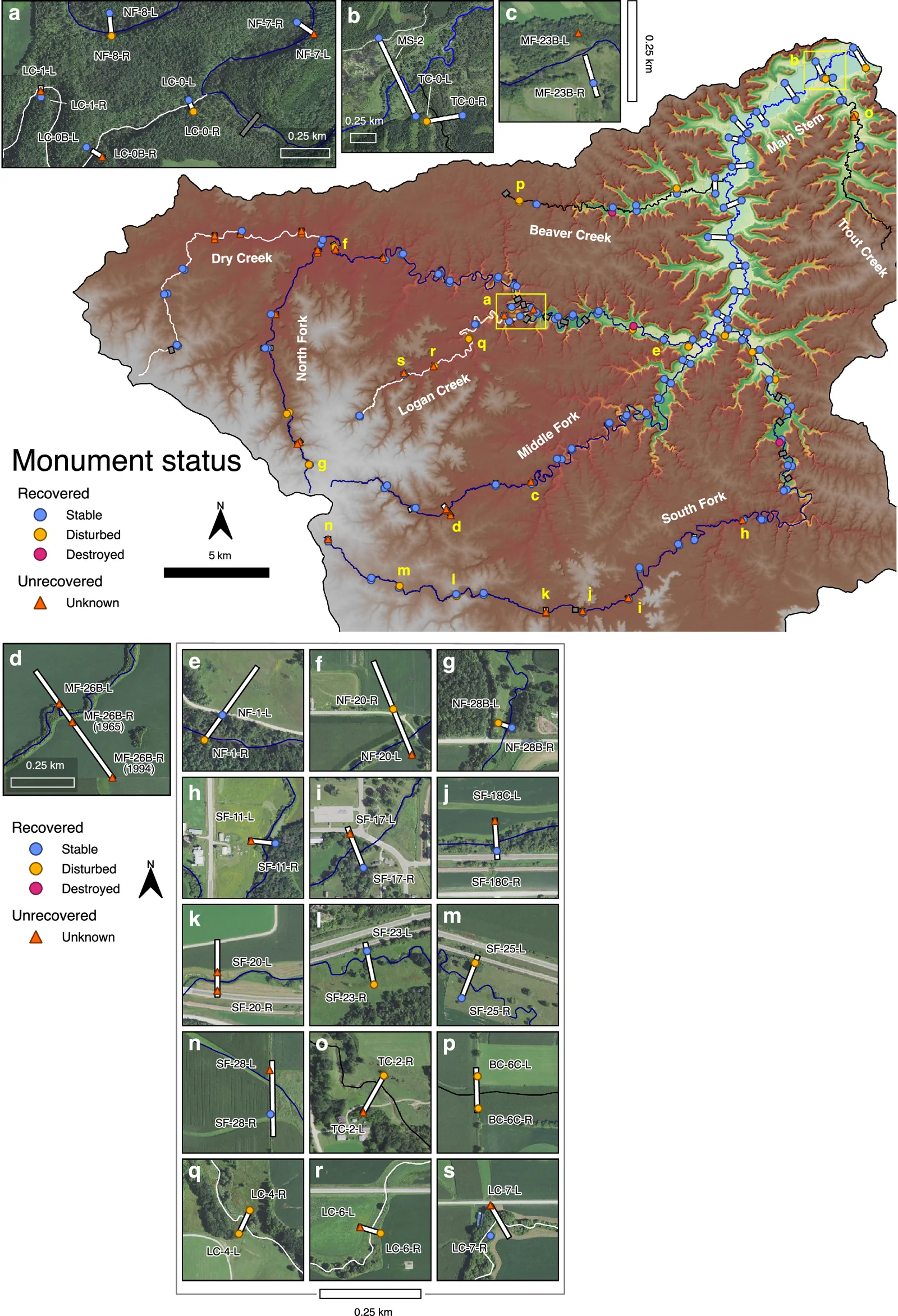
SCS–USGS–ARS–NRCS stream-and-valley-sedimentation-survey transect monuments surveyed by NRCS–Pennsylvania State University personnel and affiliates in the Whitewater River Valley between 2008 and 2014. The basin map displays the location of 189 survey monuments that tangibly marked the 94 active floodplain survey transects (white lines) when they were established^19,65,86,88,91^. Monuments are symbolized by their last field recovery status (2008–2014). Those that were physically recovered (found) are indicated by a circle and those that were unrecovered are indicated by a triangle. Monuments are further distinguished by their stability status, which speaks to their utility as an elevation benchmark. Monuments that lacked visible evidence of soil or marker disturbance are assumed to be vertically stable and colored blue. Disturbed monuments are colored yellow. Those that were confirmed destroyed are a magenta. All unrecovered monuments have an unknown stability status (orange). Insets (yellow boxes/letters) are provided where distinguishing individual monument status is difficult due to the crowding of map elements at the basin scale^98^: (**a**) North Fork–Logan Creek confluence, (**b**) Main Stem–Trout Creek confluence (TC-0), (**c**) MF-23B, (**d**) MF-26B, (**e**) NF-1, (**f**) NF-20, (**g**) NF-28B, (**h**) SF-11, (**i**) SF-17, (**j**) SF-18C, (**k**) SF-20, (**l**) SF-23, (**m**) SF-25, (**n**) SF-28, (**o**) TC-2, (**p**) BC-6C, (**q**) LC-4, (**r**) LC-6, and (**s**) LC-7.

Doug Christianson (NRCS), a 1994 surveyor, revisited the SCS–USGS transects about 15 years after the last survey and began a GNSS survey of the range monuments (Fig. 8). Christianson contributed greatly to a similar effort conducted during and after the 1994 survey, however, issues with coordinate accuracy and dataset completion made followup work with improved technology desirable^19^.

Lawrence Svien incorporated this GNSS survey into his graduate research conducted at Pennsylvania State University^19^. Though officially concluded in 2012, the survey continued into 2014 on a volunteer basis. Local and also employed by the NRCS, he would have been familiar with the Whitewater Watershed sedimentation surveys and also valued the preservation of the transect monuments for future study.

Christianson and Svien surveyed the monument and benchmark coordinates with recreational-, mapping-, and survey-grade receivers. Increasing in grade (and typically, accuracy), the receivers used were the Garmin GPSMAP 76, Garmin Oregon 650t, Trimble GeoXT (2008 and 6000 series), and Trimble R8. The exact differential corrections employed were largely receiver dependent, but included the use of a Virtual Reference Station (VRS), the National Differential Global Positioning System (NDGPS), and the Wide Area Augmentation System (WAAS)^19^. Depending on the receiver–correction combination, coordinates have an estimated accuracy of 0.25–5 m^59–64^. The projected coordinate reference system (CRS) for these data is NAD83 / UTM zone 15 N (EPSG: 26915).

Christianson and Svien successfully collected coordinates for all 94 monumented transects. When a monument was located, they followed receiver-dependent procedures to survey their coordinates. Three previously unrecovered monuments were located—MS-11B-S (1939), and BC-6C-R and -L (both 1965). Three more were recovered but confirmed destroyed as they were removed from the earth—NF-2-L, SF-4-L, and BC-3-R. Including the 18 other monuments left unrecovered and unreplaced in 1994, seven more could not be located during this GNSS survey, which left 25 of the original 187 monuments, or their replacements, unaccounted for by 2014. Coordinates for the unrecovered monuments and those that were confirmed destroyed were surveyed at their approximate transect locations as indicated by the field notes^19^. Lastly, the MF-26B-R coordinate did not coincide with the 1994 monument position, but rather the unrecovered 1965 monument position.

### 2021–2025: University of Minnesota Twin Cities

In 2021, University of Minnesota (UMN) researchers led by Jimmy J. Wood began to compile the Whitewater Watershed stream and valley sedimentation data for publication (this study). Lawrence Svien directly provided scans of the historical government survey materials (1939–1994) and his and Christianson’s original monument coordinate files (2008–2014). Original sedimentation survey records from 1939 and 1965 are likely stored at one of the U.S. National Archives and Records Administration’s facilities. Heynen^40^ notes that, by 1975, the SCS transferred maps, cross-sections, and field notebooks for these Whitewater projects to the National Archives’ Washington and Upper Mississippi Regional Offices. In 1993, the ARS also described plans to send materials to the National Archives’ Southeast Regional Office, though our records contain no confirmation that this was completed (Murphey, J. B., personal communication, April 7 & October 22, 1993). We drew the raw data for our study from these documents. Svien’s insights also led us to the *Stanley Trimble Papers* Special Collection (LAX Mss 2013/02) at the University of Wisconsin–La Crosse Murphy Library; primary source materials (including original data) related to the SCS–USGS–NRCS Whitewater surveys comprise a significant volume. A group of (under)graduate students digitized most of the records in this collection.

Two undergraduates and their graduate supervisor entered the target field data from the boring, elevation, and coordinate surveys into conventional spreadsheets. The target data were boring survey LS thicknesses, borehole depths, and measurement stations; elevation survey elevations and measurement stations; coordinate survey monument Northing and Easting values; and relevant survey, monument, and transect metadata^65^.

**Fig. 9:**
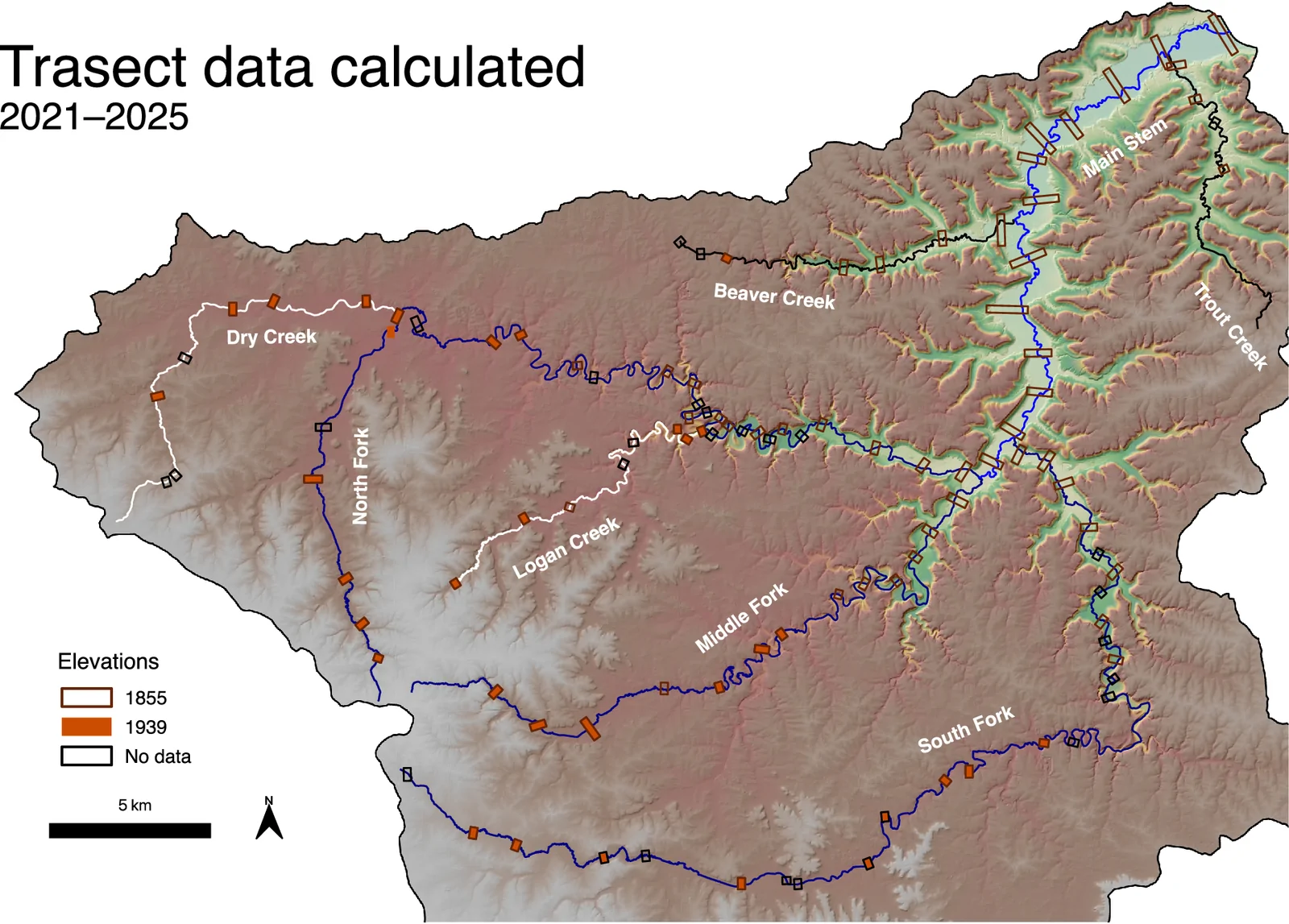
Transect data calculated between 2021 and 2025 by University of Minnesota Twin Cities (UMN) researchers for the historic stream and valley sedimentation survey dataset from the Whitewater River Valley. To augment the temporal range of this dataset, we calculated transect surface elevations for 1855 (the pre-Euro-American floodplain) by subtracting boring LS thicknesses from their temporally coincident surface elevations. Where applicable, we were then able to calculate surface elevations for 1939 by adding the older LS thicknesses to the 1855 elevations^65,86,88,91^. The thirty transects that have “No data” either lack the input data needed to carry out these operations or are subject to exceptions outlined in **Methods**.

We also created original transect elevation datasets via procedures outlined by Happ^31^ (Fig. 9). Assuming that the base of LS accumulation temporally coincides with the introduction of Euro-American agriculture, we reconstructed the pre-Euro-American floodplain surface by subtracting LS thicknesses from ground elevations at coincident transect stations. By necessity, the borehole and elevation data used in this procedure came from the same survey—1939 or 1965. Recall that transect boring and elevation stations were typically measured by different methods—paced vs. taped/chained. These methods yielded different measurements so we converted each boring station to the coincident elevation station to appropriately perform the calculations. Most of these conversions were explicitly provided in the field notes of prior surveyors as station equivalences. Otherwise, they were inferred from station and topography notes. Uncertainties for subtractively reconstructed elevations equal those of the source elevation data. They do not consider uncertainty introduced from the boring measurements and so are minimum values. We set the date of the resultant pre-Euro-American surface profiles to 1855, which we consider a conservative latest estimate for the widespread start of Euro-American agriculture in the basin as it coincides with the first official land sale.

Most of the 94 transects have an 1855 elevation dataset except where boring data availability, our ability to determine coincident stations, or procedure exceptions precluded calculation. Because the 1939 borings on transects SF-16, -17, -22, and -23, yielded zero LS accumulation, following the above procedure with their 1965 borings and elevations yields the 1939 surface instead of that for 1855.

We also modified Happ’s^31^ procedure to reconstruct the 1939 surface for the middle and upper valley transects that lacked 1939 elevation data and were bored in 1939 and 1965. Assuming that the base of LS accumulation for the two boring sets are the same surface (1855), adding the 1939 LS thicknesses to the 1855 elevations at coincident transect stations yields the 1939 surface. Two-thirds of the transects with the requisite data now have a 1939 surface profile where none existed before. The rest of those transects were precluded by the reasons mentioned previously. An added exception included our inability to align the two boring surveys because of transect relocations in 1965.

Since the NRCS raised select transects to NGVD 29, we were able to transform their previously surveyed local elevations to this absolute datum. Assuming that a monument’s elevation has remained stable over time, and that its past local elevation is functionally equivalent to the 1994 NGVD 29 value, we added their difference to all local elevation values for SF-4, -18C, -25; TC-0, -1, -2, -3; and DC-1. Closures for these datum transformed elevations are calculated via standard error propagation procedures except where 1994 closures could not be ascertained from the field notes^65^.

A single researcher verified the entered historical and original data through visual-check and double-entry procedures, respectively^66^. We first visually compared every historical data point and their metadata to the corresponding source material. We then recalculated our original data and automated their comparison to the first result. Discrepancies noted visually by the verifier or automatically by the spreadsheet were investigated and corrections were applied where applicable. After one round of verification, we plotted the elevation data per transect to further identify erroneous points that were missed during the visual check and double entry.

With the compiled sedimentation data, we calculated mean-transect-elevation-change rates (cm/y) for various time intervals between 1855 and 1994 that are largely bounded by the historical surveys. The primary intervals we investigated were 1855–1939 (84 years), 1939–1965 (26 years), and 1965–1994 (29 years). The additional surveys of MS-11B led to further calculations for that transect over 1965–1975 (10 years), 1975–1978 (3 years), and 1978–1994 (16 years). Lastly, some transects without a 1939 dataset have a mean-transect-elevation-change rate for 1855–1965 (90 years). Mean-transect-elevation-change-rate calculations followed one of two procedures dependent upon the availability of elevation data for the time interval under investigation.

**Fig. 10:**
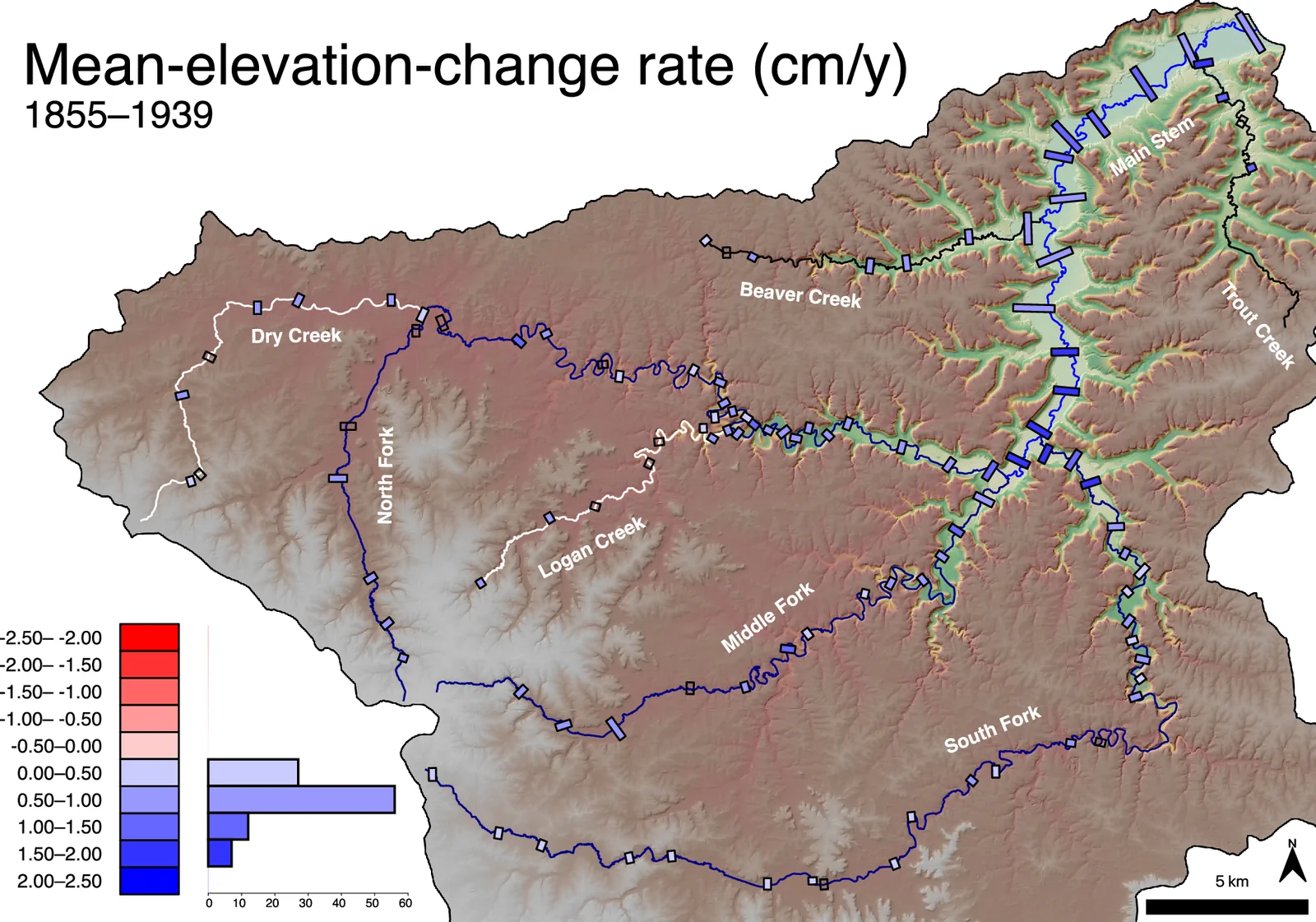
Mean-transect-elevation-change rates (cm/y) in the Whitewater River Valley between 1855 and 1939. The histogram next to the map legend displays the frequency at which the calculated rates occur—all indicate net deposition. Though floodplain sedimentation was surely severe during this time interval, this phenomenon signals limitations in our elevation-change-rate calculation methods; we cannot measure erosion from the LS thickness data. Regardless, deposition is most severe in the lower valley, particularly at the confluences of the three Forks and in the lower Main Stem. Change-rate uncertainties for this period are + /- 0.000–0.16 cm/y^65,86,88,91^.

**Fig. 11:**
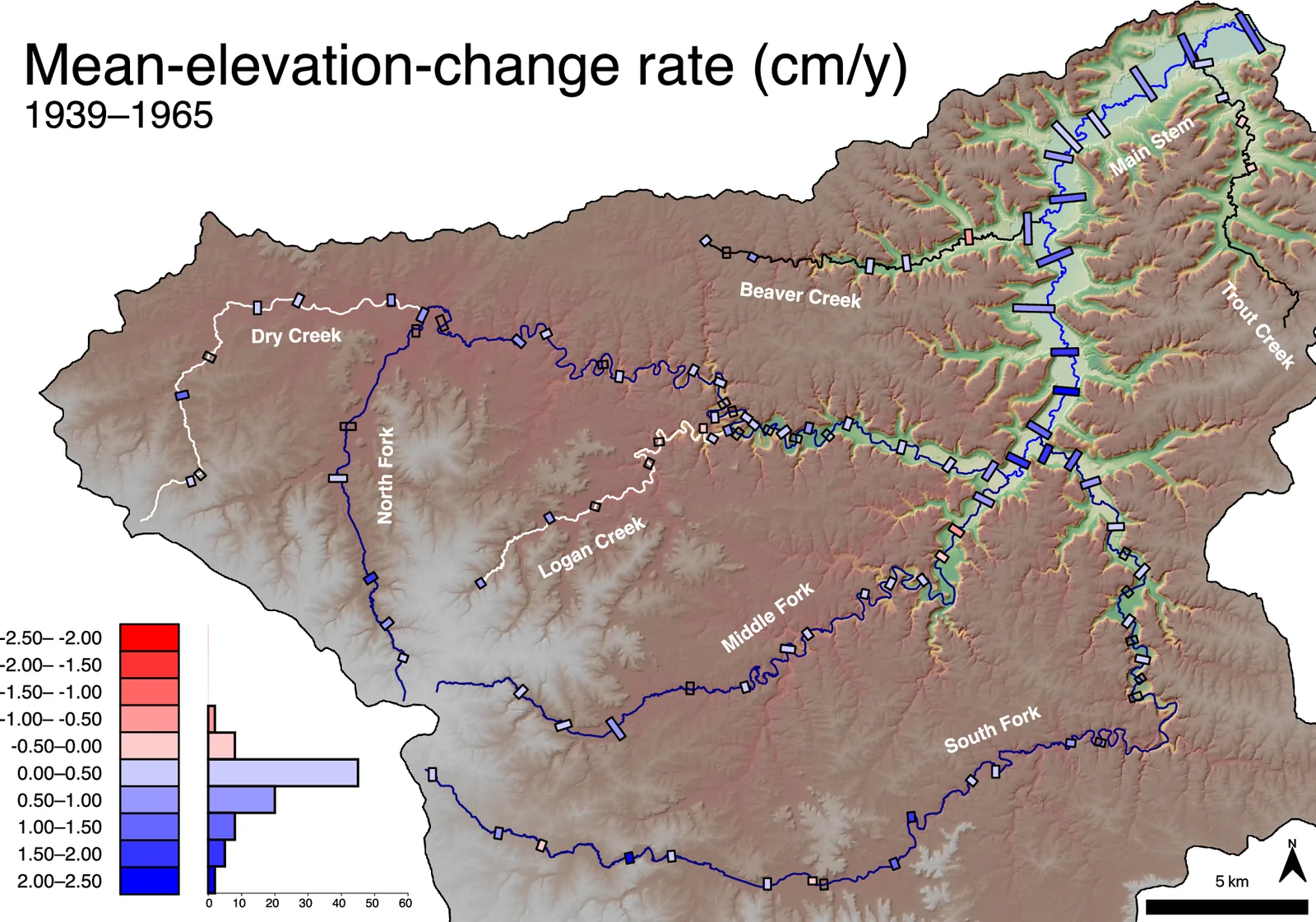
Mean-transect-elevation-change rates (cm/y) in the Whitewater River Valley between 1939 and 1965. Net erosion, now detectable, mainly occurs in the basin’s middle and upper valleys. Deposition is still generally most severe in the lower valley. Change-rate uncertainties for this period are + /- 0.000–0.41 cm/y^65,86,88,91^.

**Fig. 12:**
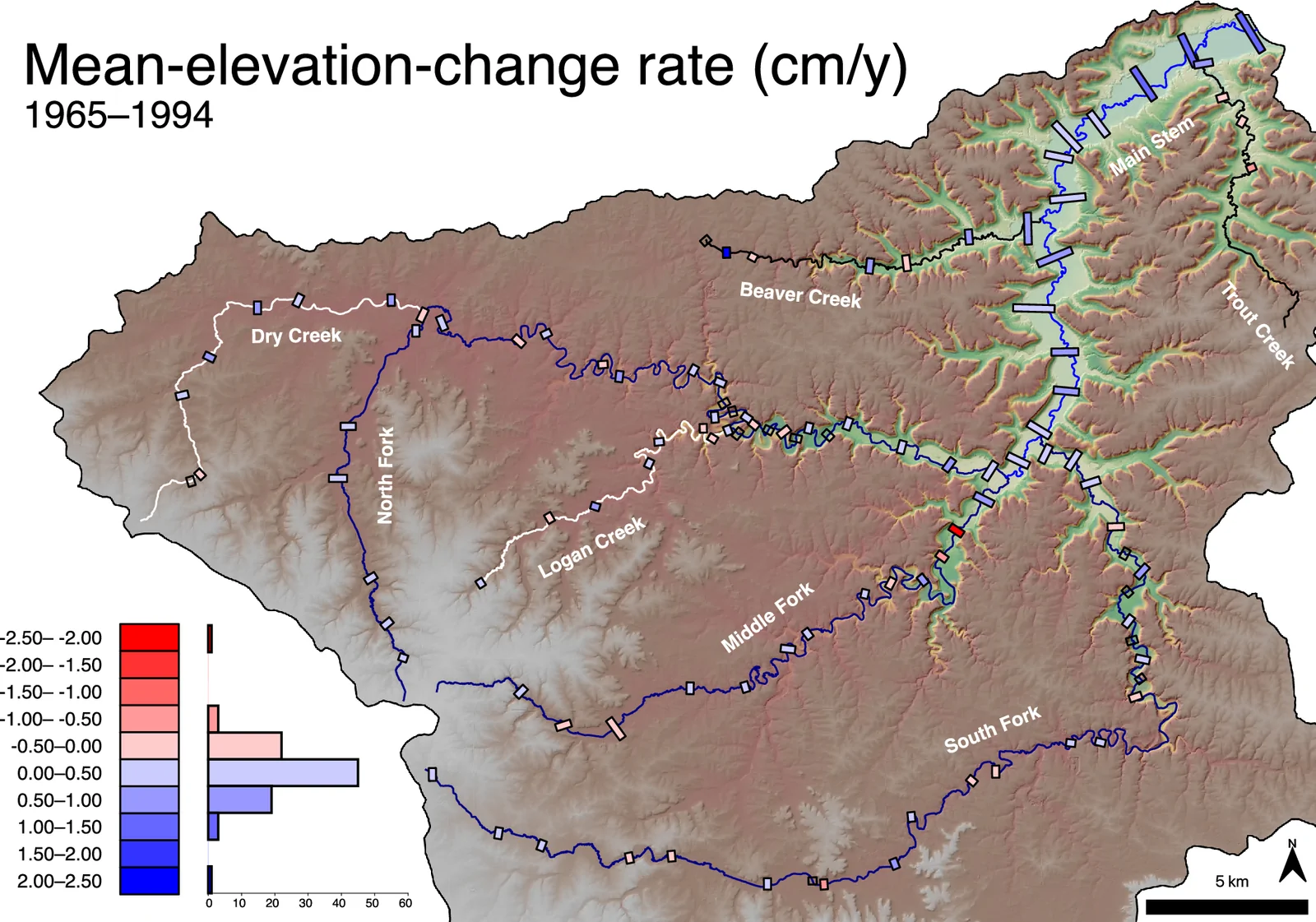
Mean-transect-elevation-change rates (cm/y) in the Whitewater River Valley between 1965 and 1994. The magnitude of both net deposition and net erosion generally decrease across the basin. More transects exhibit net erosion than previous periods, though it is still concentrated in the middle and upper valleys. Change-rate uncertainties for this period are + /- 0.000–1.00 cm/y^65,86,88,91^.

Our preferred calculation method utilizes two elevation datasets. When available, we first resampled both elevation sets (in 0.1 horizontal foot increments via linear interpolation) to overcome the fact that elevations were not measured at the same transect stations between surveys and thus enable their direct comparison. At each interpolation point we calculated the elevation-change rate by dividing the elevation change between the newer and older surfaces by the length of the time interval. From the interpolation-point rates, we calculated the mean-elevation-change rate over the entire transect. Mean-transect-elevation-change-rate uncertainties are propagated from the closure errors of the surface profiles used. For surveys with both local and NGVD 29 elevations, we calculated rates with the absolute datum values except when this maximized the rate uncertainty, in which case we used the local data. Figures 10–12 show the mean-transect-elevation-change rates for 1855–1939, 1939–1965, and 1965–1994.

Some individual interpolation-point-elevation-change rates were excluded from the mean-transect-rate calculation for various exceptions. Some surface profiles are effectively discontinuous due to higher-than-average sample spacings or gaps. For example, the 1994 survey of MS-3 has a sampling gap of about 270 m on its left side. We consider this too large to reasonably interpolate through and entirely omit rates calculated in that 270 m section from the mean-1965-to-1994-rate calculation. Some interpolated profiles are also truly discontinuous between points where zero LS was measured along a transect. Transect SF-2 lacked LS over a 40 m stretch along the right bank of the river in 1939, which is excluded from the mean-1855-to-1939-rate calculation. Mean rates for 1855–1939 exclude all negative interpolation point rates indicating erosion since we have no knowledge of the actual, continuous geometry of the floodplain surface in 1855 and therefore cannot directly compare it to 1939. Similarly, when using our calculated 1939 surface profile data, we omit negative interpolation-point rates for the 1939–1965 rate calculated in the river channel as we lack channel geometry data for the earlier year. For calculation exception zones that span only part of a transect, we have provided the start and end elevation stations where we apply the exception.

Without two surface profiles, we calculated mean-transect-elevation-change rates from the auger-boring data. For the 1855–1939 interval, the sedimentation rate at each borehole is calculated by dividing the LS thickness by the length of the time interval. For transects with no 1939 elevation data, the 1939–1965 rate was calculated at each borehole by first subtracting the 1939 LS thickness from that of 1965, and dividing the result by the length of the time interval. Because no LS was recorded in the 1939 boring surveys of SF-16, -17, -22, and -23, their 1855–1939 mean-transect-elevation-change rates are assumed to be 0 cm/y.

Accompanying our mean-transect-elevation-change rates are the results of a statistical analysis to gauge the significance of their change over time^65^. Test samples are grouped by time interval—(1) 1855–1939, (2) 1939–1965, and (3) 1965–1994—and compared via Analysis of Variance (ANOVA). To select the appropriate statistical testing procedure, we verified our sample groups’ compatibility with ANOVA’s underlying assumptions of independence, normality, and collective homoscedasticity (variance equality)^67^. Each sample group is independent because rates from one are not used to calculate rates from another. Normality and homoscedasticity were investigated through the Kolmogorov–Smirnov^68^ and Brown–Forsythe^69^ tests, respectively. Their *p*-values and an invocation of the Central Limit Theorem^70,71^ enable the use of the conventional one-way ANOVA omnibus test followed by Tukey’s HSD (honestly significant difference) post-hoc pairwise comparison test^72^. Both test the null hypothesis (*H*_*o*_) that mean-transect-elevation-change rates between time intervals are equal and the alternate hypothesis (*H*_*a*_) that they are not equal. All statistical tests were carried out at the 0.05 level of significance (*α*).

Lastly, we digitized the bulk of the transect elevation, monument, and mean-transect-elevation-change-rate data for geospatial analysis and visualization. We digitized all transect elevation data points through coordinate geometry (COGO) calculations from one of the transects’ monument positions, usually the one used as the survey-start point for the transect^73^. For monuments shifted off of the transect, their displacement was compared to their estimated coordinate accuracy prior to elevation digitization; when the displacement was greater than that accuracy, the points were shifted accordingly, away from the monuments. We digitized the monuments directly from their surveyed coordinates and manual COGO operations for the 1994 replacement of MF-26B-R. All 107 transect lines were also digitized with their corresponding mean elevation change rates. Where applicable, the lines were manually digitized onto the 1994 elevation data. The positions and orientations of the retired SF-19, BC-7, and DC-9 were modeled after annotated aerial photos and topographic maps^74–76^. The North and South Fork reservoir silting transect positions were approximated from data table information and, in the absence of range bearings, we set their orientations to be orthogonal to the river. Retired transect length is representative of their field surveyed extents.

## Data Records

**Fig. 13:**
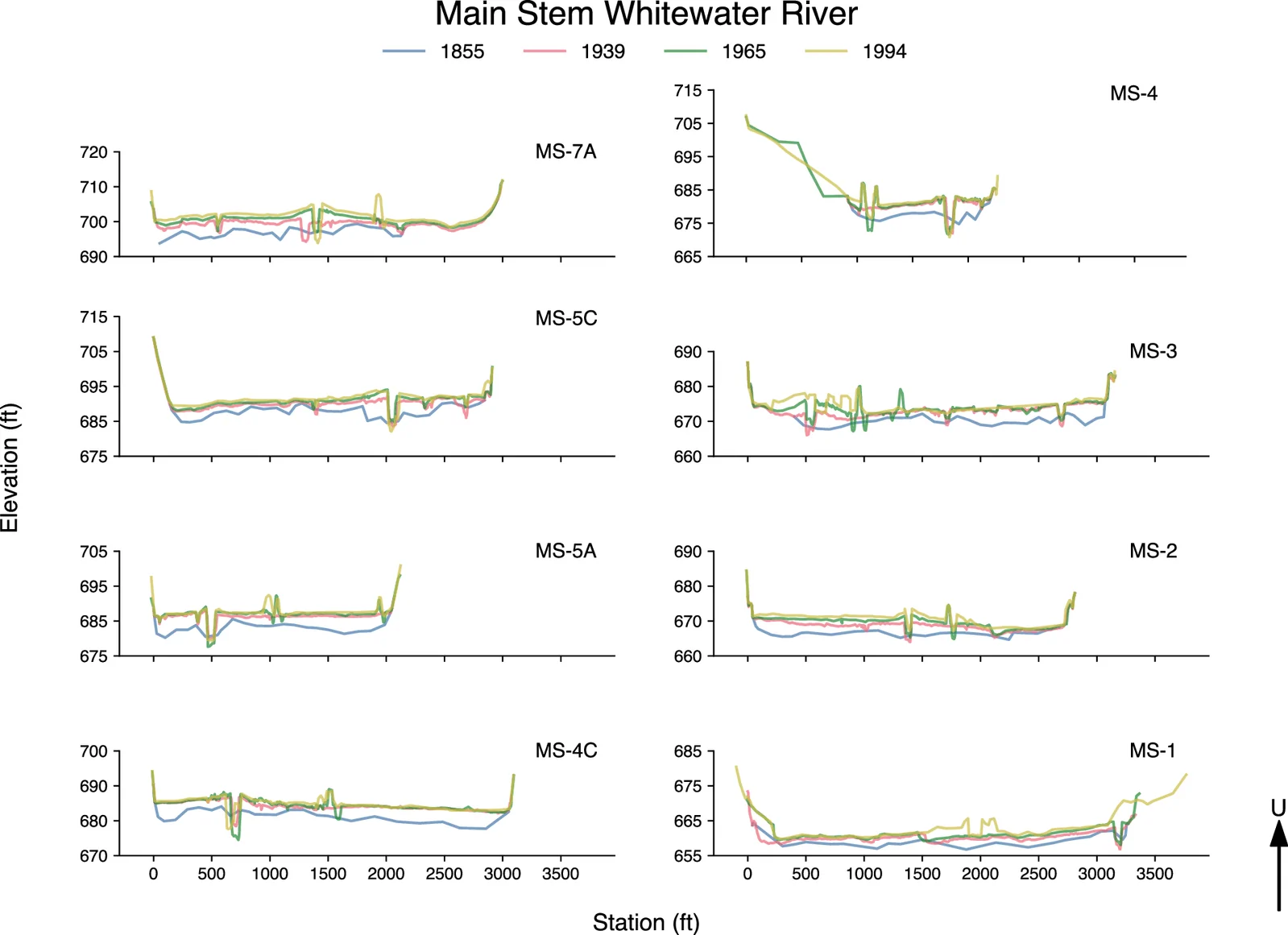
Main Stem Whitewater River cross-sections for transects MS-1–MS-7A. The arrow points upstream from MS-1 and -4C. The vertical exaggeration is 30^65^.

**Fig. 14:**
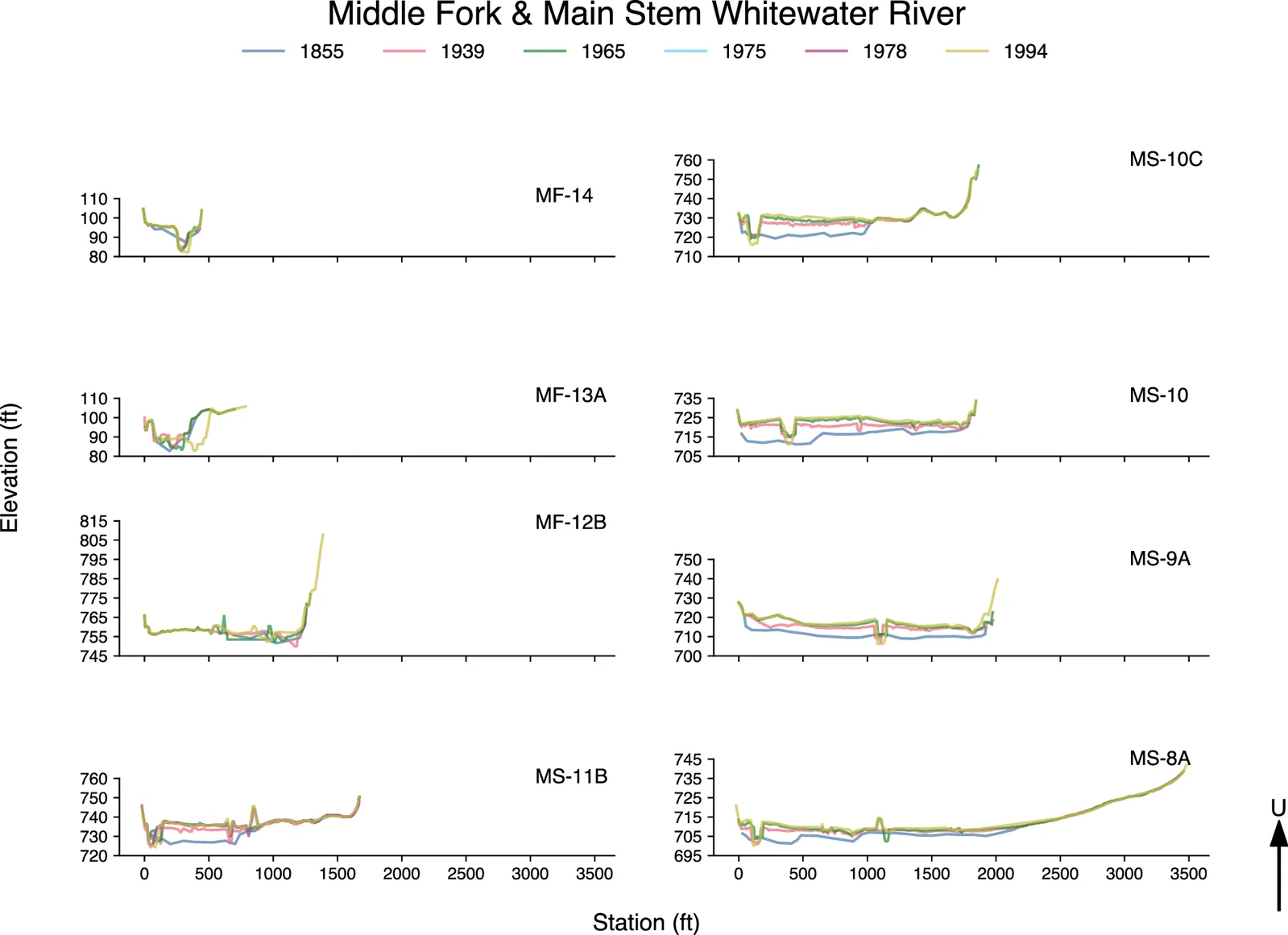
Middle Fork & Main Stem Whitewater River cross-sections for transects MS-8A–MF-14. The arrow points upstream from MS-8A and -11B. Transect MS-11B was the only range surveyed in 1975 and 1978. The vertical exaggeration is 15^65^.

**Fig. 15:**
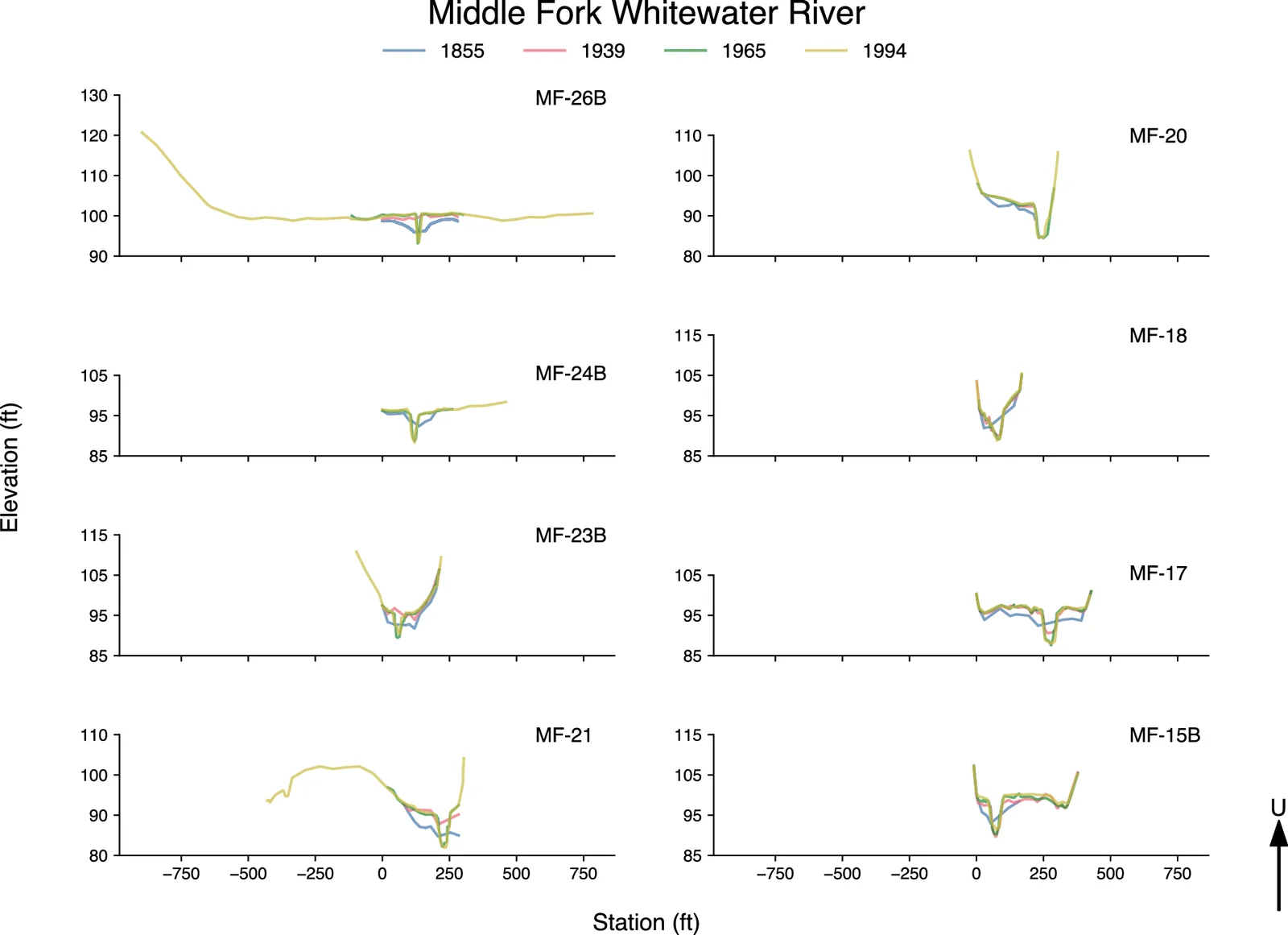
Middle Fork Whitewater River cross-sections for transects MF-15B–MF-26B. The arrow points upstream from MF-15B and -21. The vertical exaggeration is 15^65^.

**Fig. 16:**
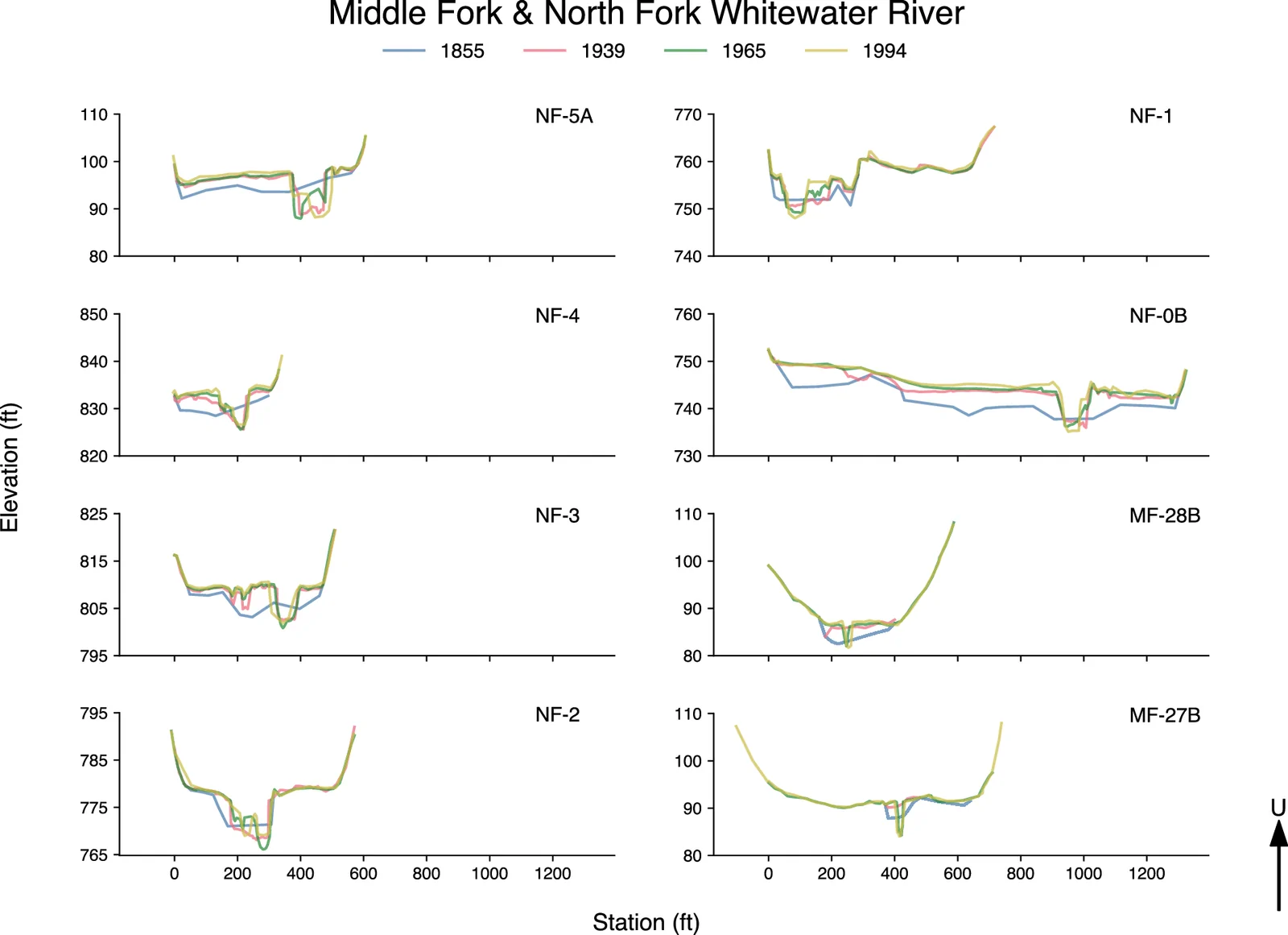
Middle Fork & North Fork Whitewater River cross-sections for transects MF-27B–NF-5A. The arrow points upstream from MF-27B, and NF-0B and -2. The vertical exaggeration is 15^65^.

**Fig. 17:**
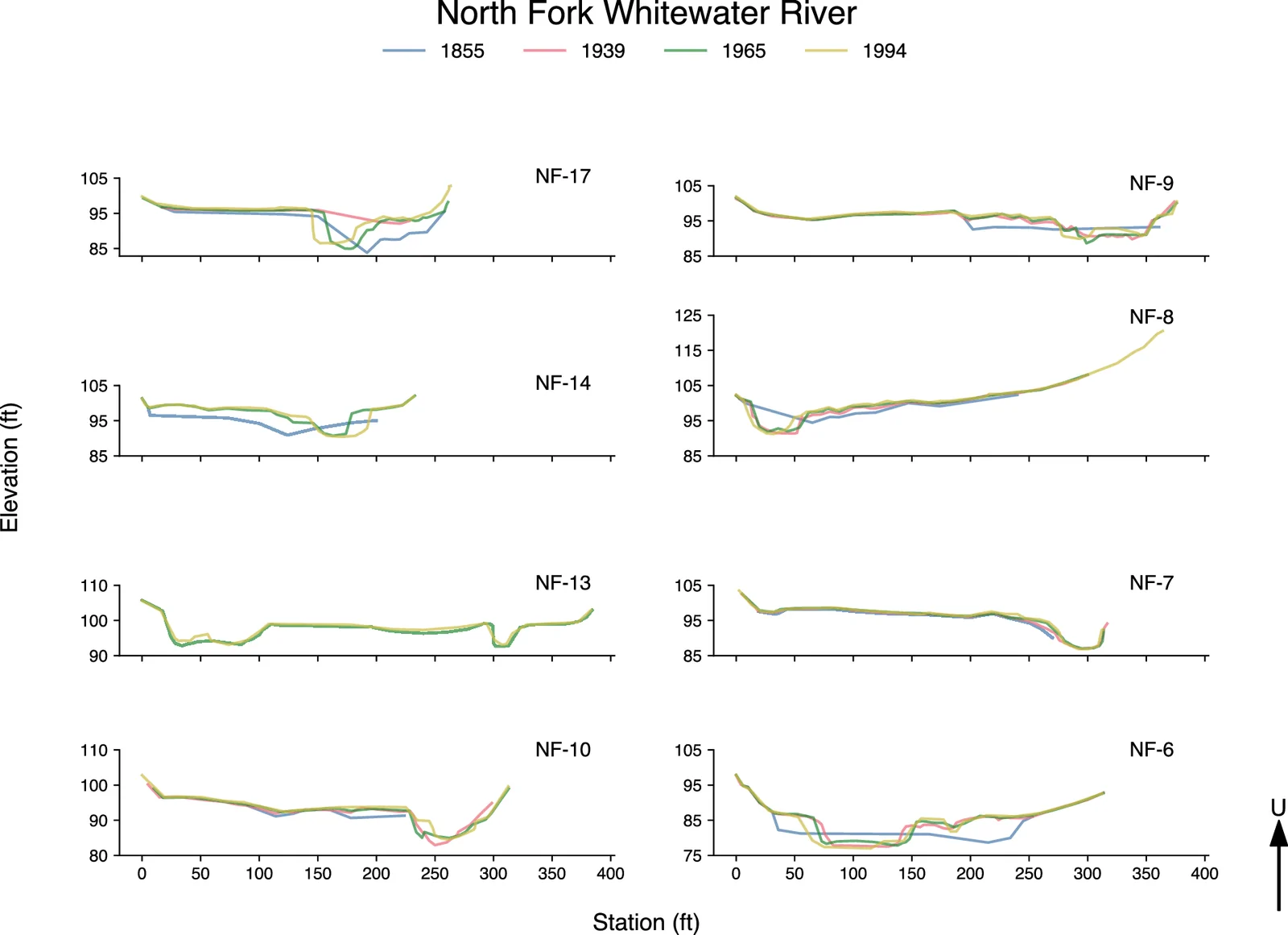
North Fork Whitewater River cross-sections for transects NF-6–NF-17. The arrow points upstream from NF-6 and -10. The vertical exaggeration is 3^65^.

**Fig. 18:**
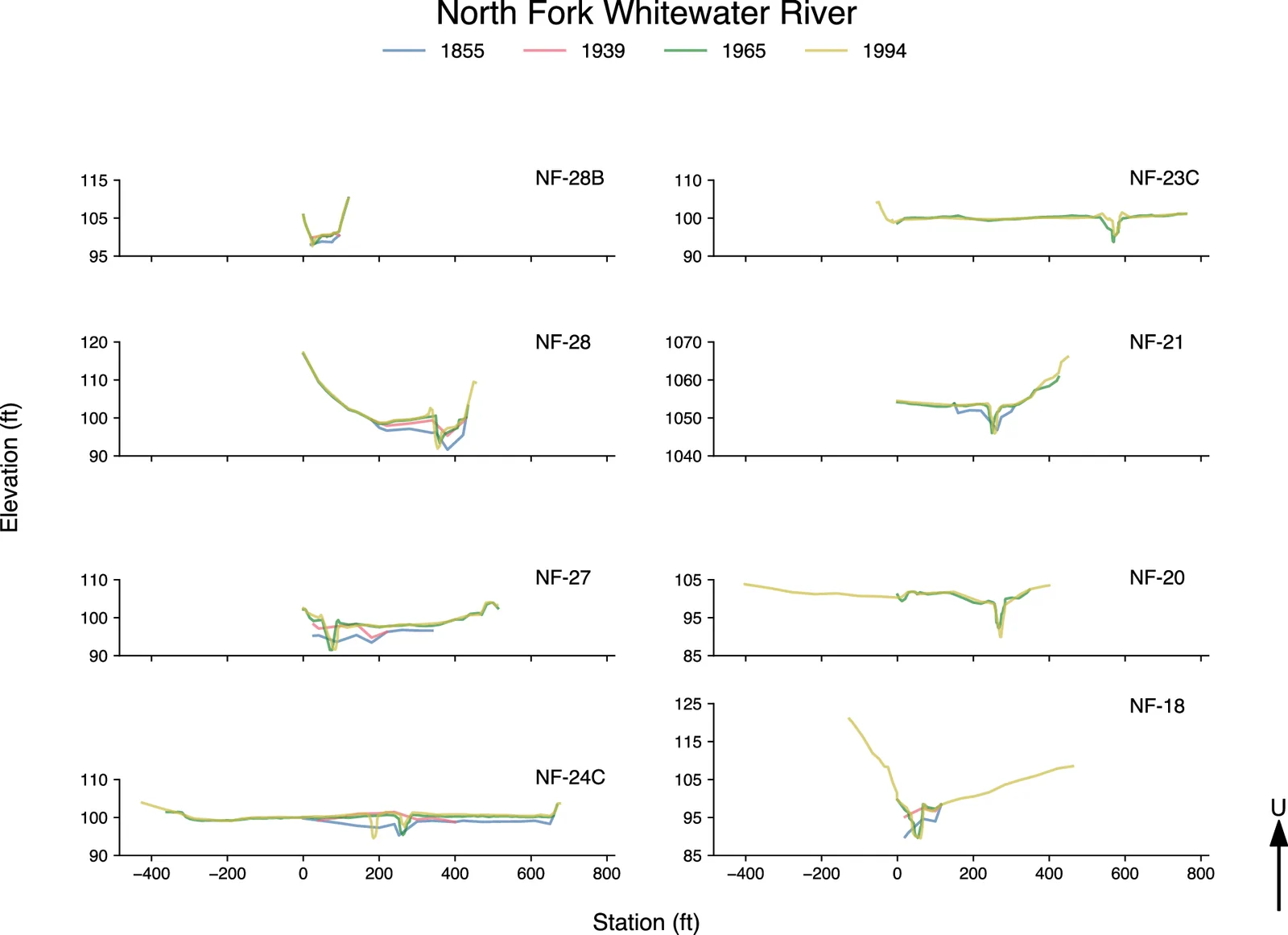
North Fork Whitewater River cross-sections for transects NF-18–NF-28B. The arrow points upstream from NF-18 and -28B. The vertical exaggeration is 10^65^.

**Fig. 19:**
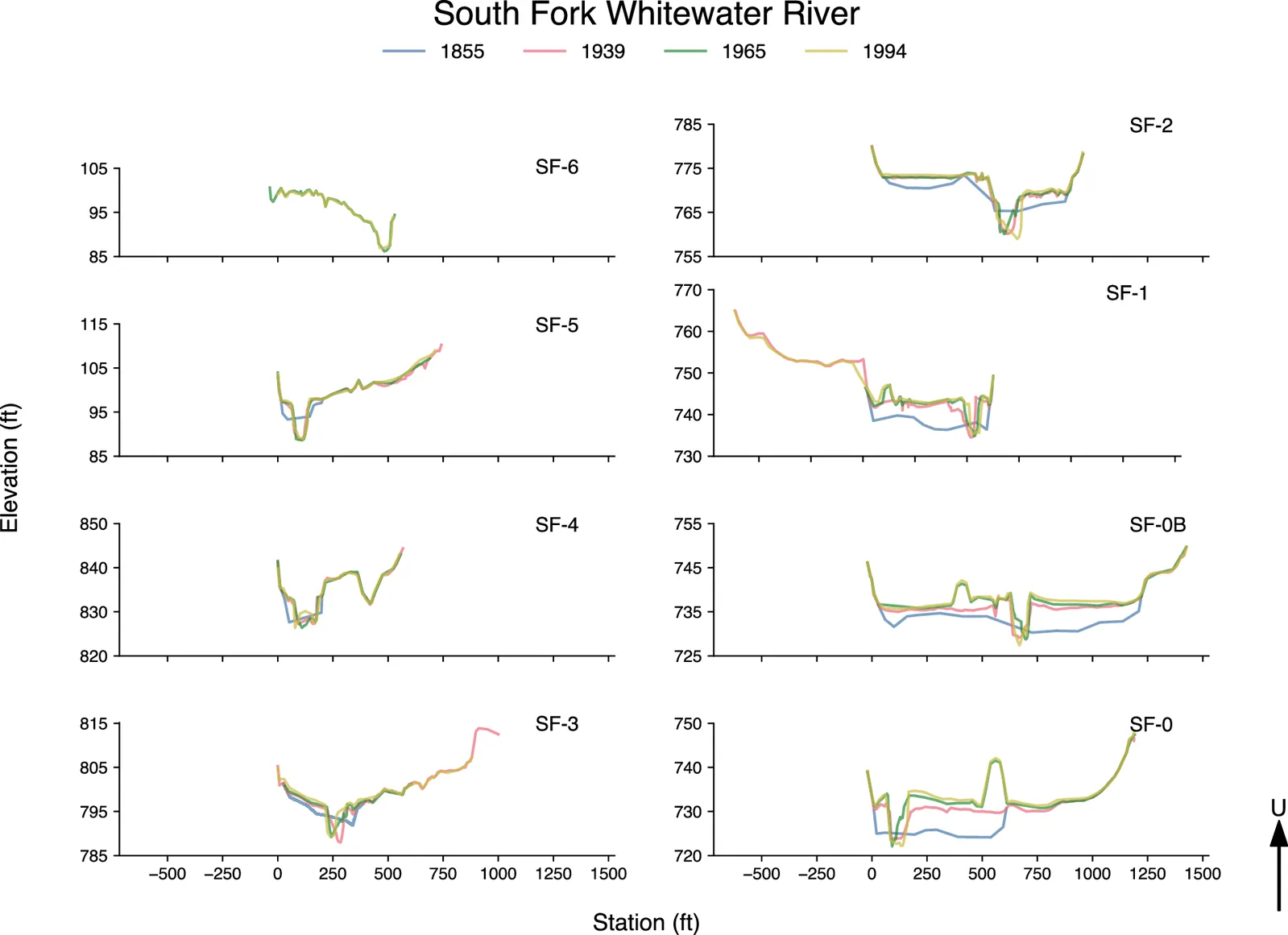
South Fork Whitewater River cross-sections for transects SF-0–SF-6. The arrow points upstream from SF-0 and -3. The vertical exaggeration is 20^65^.

**Fig. 20:**
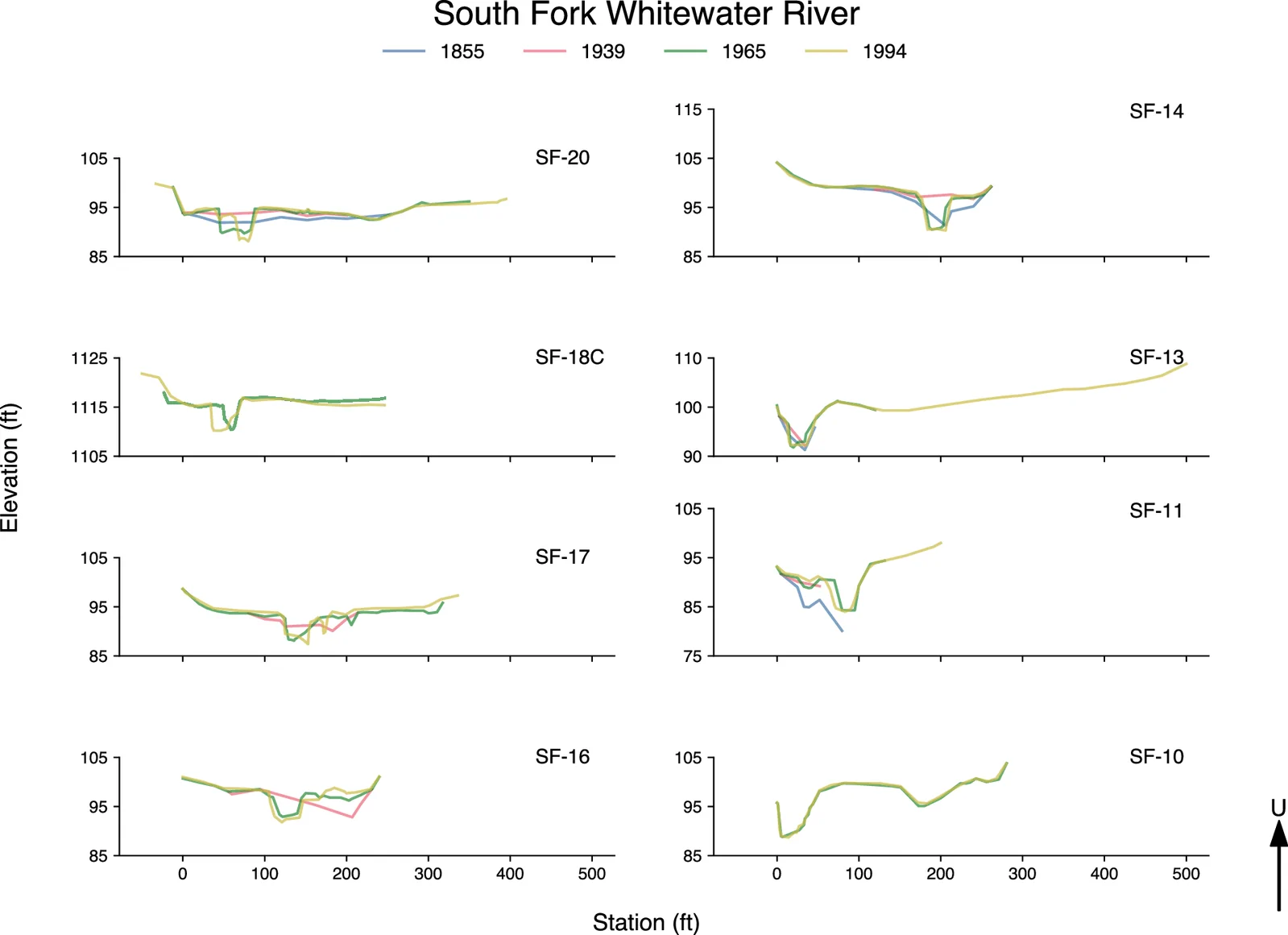
South Fork Whitewater River cross-sections for transects SF-10–SF-20. The arrow points upstream from SF-10 and -16. The vertical exaggeration is 6^65^.

**Fig. 21:**
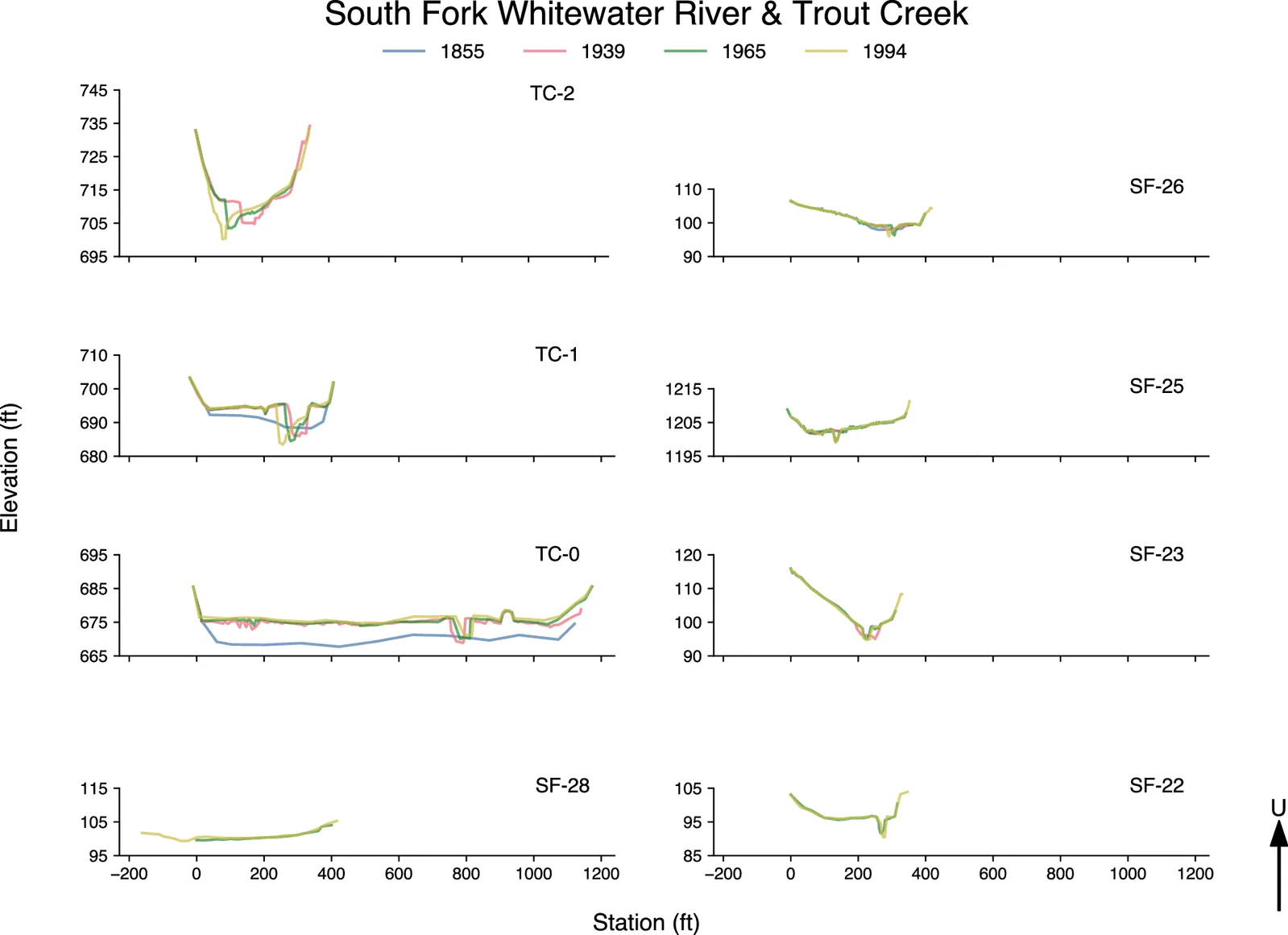
South Fork Whitewater River & Trout Creek cross-sections for transects SF-22–TC-2. The arrow points upstream from SF-22 and -16, and TC-0. The vertical exaggeration is 10^65^.

**Fig. 22:**
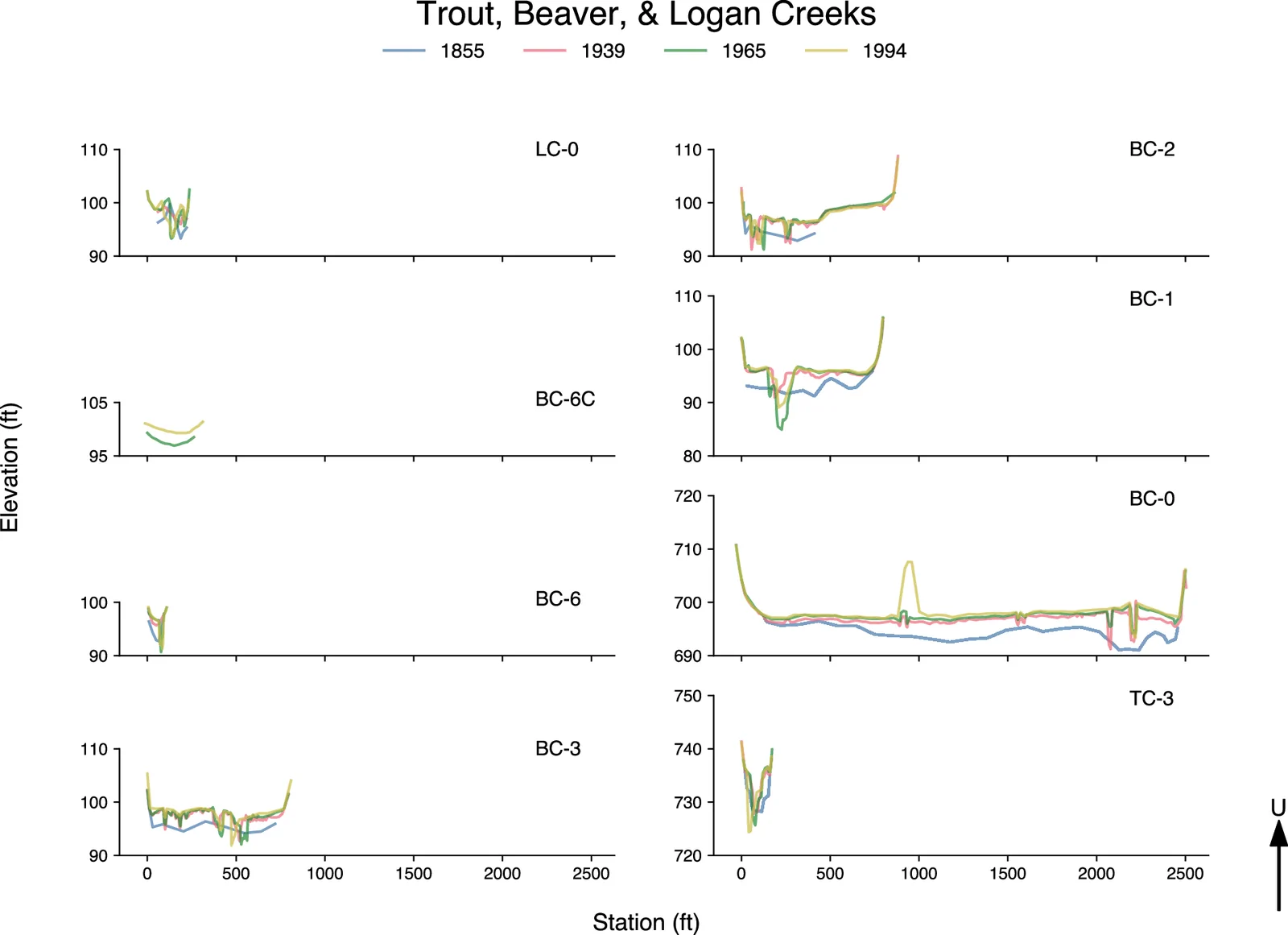
Trout, Beaver, & Logan Creek cross-sections for transects TC-3–LC-0. The arrow points upstream from BC-0 and LC-0. Transect TC-3 is Trout Creek’s furthest upstream range. The vertical exaggeration is 30^65^.

**Fig. 23:**
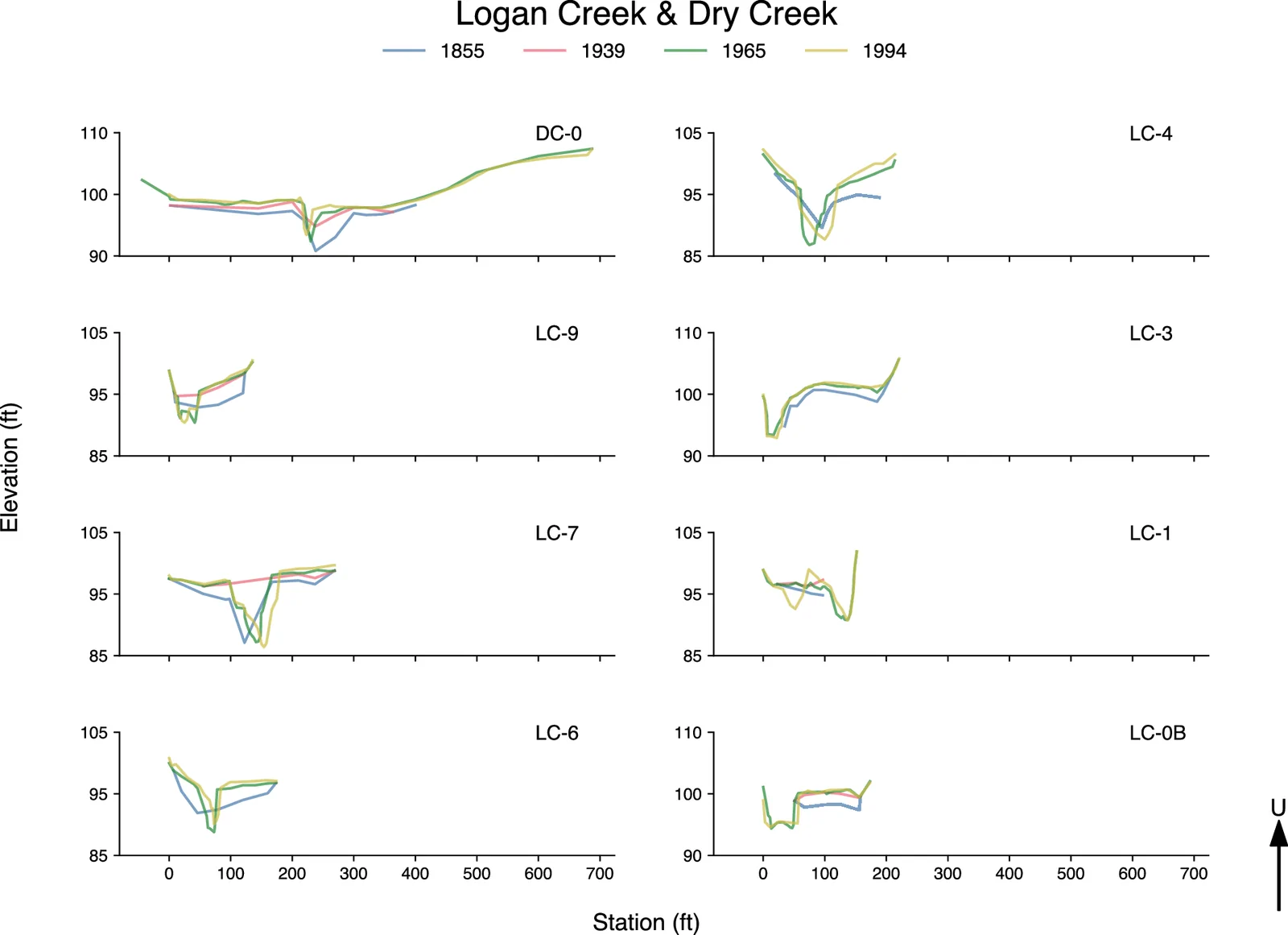
Logan Creek & Dry Creek cross-sections for transects LC-0B–DC-0. The arrow points upstream from LC-0B and DC-0. The vertical exaggeration is 10^65^.

**Fig. 24:**
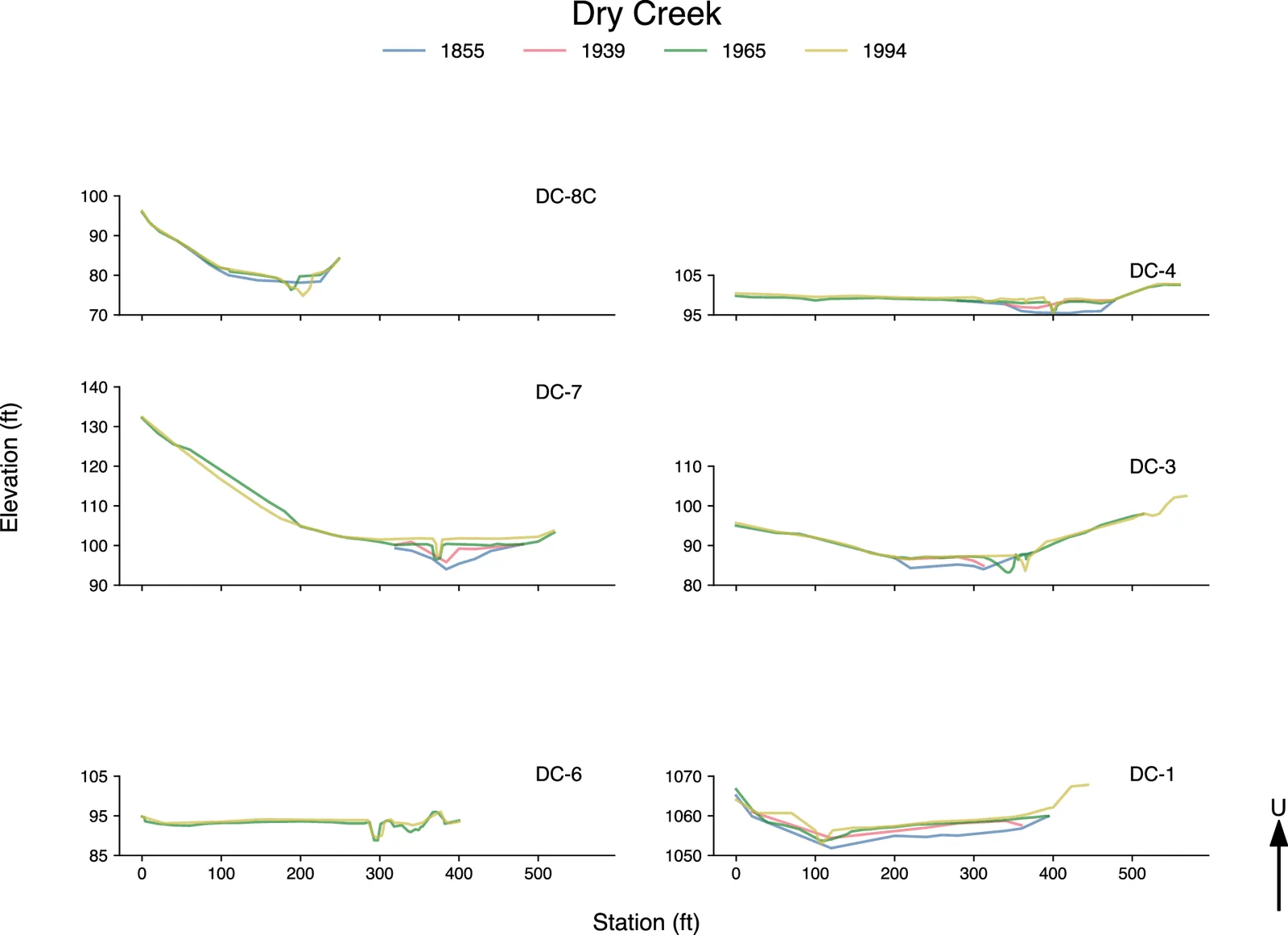
Dry Creek cross-sections for transects DC-1–DC-8C. The arrow points upstream from DC-1 and -6. The vertical exaggeration is 5^65^.

The stream-and-valley-sedimentation-survey data for the Whitewater River Watershed, collected or calculated between 1939 and 2025 is stored in the Data Repository for the University of Minnesota (DRUM)^65^. To effectively distinguish the many data/measurement types associated with this collective study, we have separated the data into eight spreadsheets: (1) transect (meta)data, (2) monument (meta)data, (3) subsurface LS thicknesses, (4) surface elevations, (5) monument coordinates, (6) mean-transect-elevation-change rates, (7) rate-calculation exception zones, and (8) rate-comparison statistical tests. The digitized stream-and-valley-sedimentation-survey data are available in a GeoPackage containing eight layers: (1) transect lines; (2) monument points; and surface-elevation points for (3) 1855, (4) 1939, (5) 1965, (6) 1975, (7) 1978, and (8) 1994. Detailed descriptions of each file and their corresponding data can be found in the READMEs within the data repository^65^. Figures 13–24 display the elevation data, in cross-section, of all 94 transects.

## Technical Validation

### Field data

**Fig. 25:**
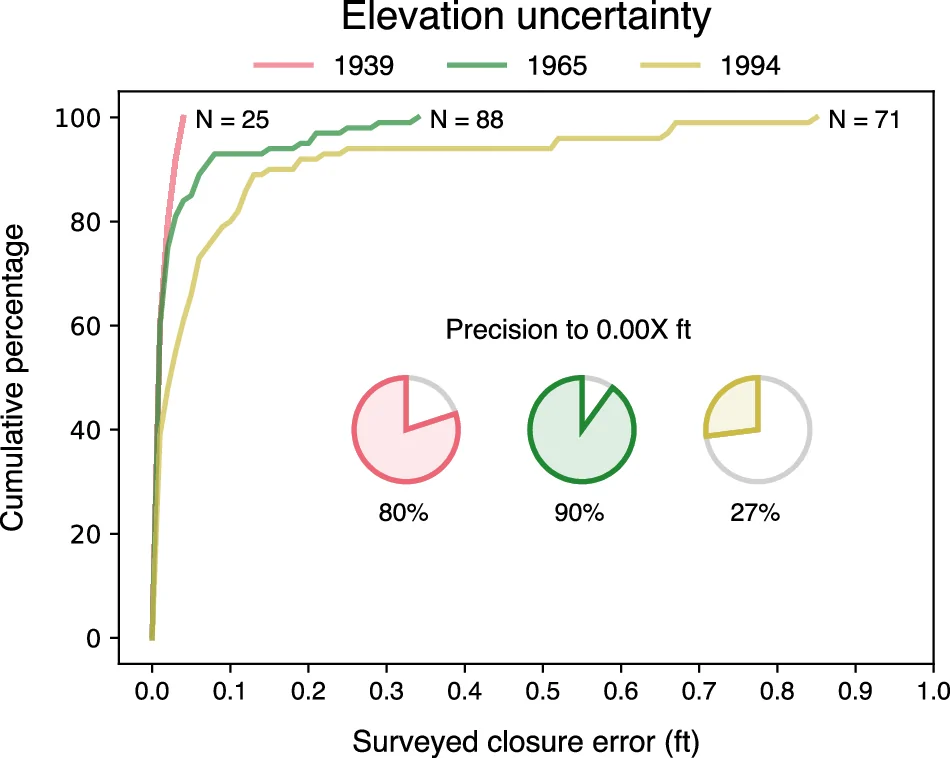
Surveyed elevation uncertainties (closure errors) measured by field surveyors in 1939, 1965, and 1994. The cumulative percentage distributions of closure-error magnitudes (lines) display the range and membership percentage of uncertainties attained per basin survey. Closure-error sample size is denoted by N; the value of N does not indicate a one-to-one relationship between the set of closure errors and the set of applicable transects. The pie charts display the proportion of closure errors measured to the thousandths of a foot. In general, closure error accuracy decreased from 1939 to 1994 as the proportion of larger errors and their maximum values increased. The maximum closure error magnitudes recorded in 1939, 1965, and 1994 were 0.036 ft (1.1 cm), 0.340 ft (10.4 cm), and 0.85 ft (26 cm), respectively. Closure error precision was high in 1939 and 1965, with 80% and 90% recorded to the third decimal place, respectively. Sixty percent of closures for both years were also within 0.010 ft (3.0 mm) from the expected standard. Both of these uncertainty-set traits dropped significantly in 1994 to 27% and 39%, respectively^65^.

The quality of our elevation data is quantitatively described by their estimated closure errors. Figure 25 displays the accuracy and precision of the field-surveyed closure errors from 1939, 1965, and 1994. Their cumulative percentage distributions indicate an overall increase in closure-error magnitude between 1939 and 1994; relatively large values constitute a greater proportion of the closure-error set and the maximum error increased 24-fold from 0.036 ft to 0.85 ft (1.1–26 cm). Collectively, the mean-transect-elevation-change-rate uncertainties for 1855–1939, 1939–1965, and 1965–1994 (which are dependent upon these elevation data) mirror this trend.

Despite the observed increases in closure errors over time, the 1939 and 1965 sets exhibit impressive measurement fidelity and share marked similarities in overall accuracy and precision. During both surveys, 60% of closures were within 0.010 ft (3.0 mm) of the standard elevation value. Eighty and ninety percent respectively, were measured to the thousandths of a foot (down to the tenths of a mm). These proportions decreased greatly in 1994; only 39% of closures were within 0.010 ft of the standard and only 27% were recorded to the thousandths of a foot (the rest were recorded to the hundredths).

We surmise that the similarities between the 1939 and 1965 closure errors are indicative of Dr. Happ’s central involvement in, and influence on, the first two surveys. Anecdotally, he was an exacting scientist. Trimble^43^, personally advised by Dr. Happ on his Coon Creek studies, states that “he had extremely high standards, whether it was closing a survey line [relevant here], describing a sediment core, or publishing a paper.” This exactness likely colored Dr. Happ’s expectations for those he led, too. To this point, Trimble^43^ adds that “other than himself [Dr. Happ], the only person he trusted to survey was W.E. Witzgall, an engineer-topographer who routinely closed long level lines with less than 0.001 foot (0.3 mm) error.” Both Dr. Happ and Witzgall were central participants of the first two surveys of the Whitewater Watershed when the majority of transect surveys resulted in closure errors between 0.001 ft and 0.009 ft (0.3–3 mm). In 1939, 48% of transect survey notes recorded the participants. Each list included W.E. Witzgall. In 1965, though participants were only recorded for 13% of the surveys conducted, Dr. Happ is listed for 86% of them. Their surveying likely extends past what is recorded, perhaps especially for Dr. Happ, given his role as the primary investigator and tendency to lead by example^43^.

We also surmise that closure-error magnitudes increased in 1994 primarily due to overriding project objectives and unique circumstances behind data collection. The NRCS’s data was collected primarily for input into a computer model abstracted to 40 acre (0.16 km^2^) cells, rather than input into direct sedimentation calculations, as Dr. Happ’s previous studies were. This coarser grained computation methodology may have permitted higher closure errors. Also, recall that over 50% of the 1994 transect surveys were conducted by a small group of weekend volunteers with limited-to-no agency support. Not only would this have limited the resources available to perform this work, but also the sheer amount of time that they could devote towards surveying; the time and effort to resurvey a range to minimize closure errors that breached a certain error threshold may not have been economically practical. Regardless, we are grateful for the efforts of these surveyors.

Consult the closure errors recorded in DRUM when using the elevation data^65^. All propagated errors for elevation datasets created by UMN researchers for the 1855, 1939, and 1965 years are equal to or greater than those surveyed in 1939, 1965, and 1994, by convention. See **Methods** for a description of the quality assurance measures and quality check mechanisms utilized in all data entry and verification.

### Geospatial data

We will describe the quality of our GeoPackage data layers through the Federal Geographic Data Committee’s (FGDC) metadata-standard quality elements of lineage and positional accuracy^77,78^. Recall that each layer-element’s position is ultimately derived from Svien’s monument coordinates^19^. We will take advantage of this lineage (digitization sources/methods) to perform a single positional accuracy assessment that applies to our monument points, elevation points, and transect lines. See the GeoPackage’s README for descriptions of the FGDCs three other quality components—attribute accuracy, logical consistency, and completeness—which we omit from this discussion^65,78^.

To gauge our layers’ positional accuracy, we modify the conventional National Standard for Spatial Data Accuracy (NSSDA) procedure for point-accuracy assessment towards the assessment of synthetic line features. The NSSDA provides a method to calculate the positional accuracy of “well-defined” (easily identifiable and measurable) points by comparing the deviation between a set of predicted and observed locations^79^. Typically, accuracy is reported with an RMSE and/or distribution-dependent error threshold^73^. Our monument data constitute the only layer whose elements may approximate such well-defined points; neither the elevation points nor the transect lines digitized onto them can be independently identified in the field or aerial imagery. With only a set of observed positions—the monument coordinates—we lack enough data to conduct the NSSDA assessment. We circumvent these limitations by treating each transect monument point pair as the well-defined endpoints of a line feature that traces all or part of a transect. Without an established standard to assess the positional accuracy of lines^73^, we modify the NSSDA procedure towards assessing the shape fidelity of our monument-bounded lines in terms of length and orientation preservation^80^.

**Fig. 26:**
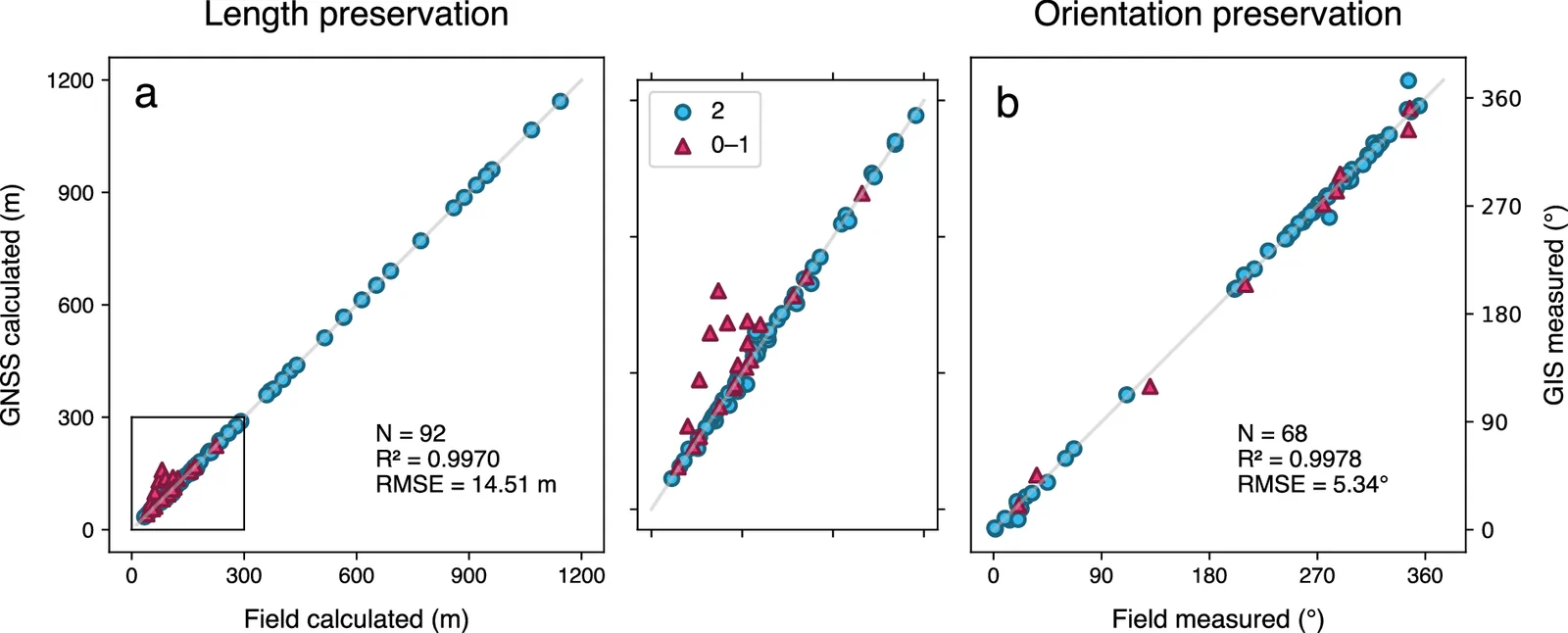
Shape-fidelity analysis of our digitized transect data products in terms of length and orientation preservation. For both quantities, we directly compare field values, those expected (or observed) to occur in reality, to those predicted by the geometry of our geospatial data layers. Both the length (**a**) and orientation (**b**) between digitized monument points display significant agreement with their corresponding field values—R^2^ values are nearly 1. The length error magnitude (RMSE) of 14.51 m is outsized by the large-magnitude residuals associated with transects where at least one monument was not recovered (red triangles), highlighted in the center inset. If they are omitted from the calculation, leaving the residuals associated with transects where both monuments were recovered (blue circles), the RMSE reduces significantly to 3.83 m. This general sensitivity to monument recovery status is not observed in the orientation error magnitude of 5.63°. Most of our residuals are small—56% of predicted lengths are within 1 m of the observed and 54% of predicted orientations are within 1° ^65^.

To investigate length preservation between monument points we compared monument station separations. For each monument pair, we calculated observed values by subtracting their field-surveyed transect stations and calculated their predicted values from the planar distance between their surveyed coordinates. Plotted together (Fig. 26a), the two separation sets display remarkable agreement with an R^2^ of 0.9970; fifty-six percent of predicted lengths are within 1 m of the observed. Despite this level of agreement, our error magnitude (RMSE) is 14.51 m. The inset plot of Fig. 26a (center) displays the region between 0 and 300 m to highlight errors yielded from transects where one, or none, of its monuments were recovered (red triangles). These are the only points that deviate significantly from one-to-one agreement. Most of their predicted, GNSS-calculated separations overestimate their observed, field-calculated separations. The resultant and relatively large magnitude residuals between these values have an outsized effect on the RMSE^73^. To demonstrate this effect, when all of these points are omitted from the RMSE calculation, it is reduced to 3.83 m. Linear shape fidelity in terms of length is preserved to a high degree for most digitized transects, especially, but not limited to, those where both monuments were last recovered.

We perform a similar comparison to investigate orientation preservation between monument points. We took the available surveyor-measured transect bearings from 1939 and 1965 and converted them to clockwise navigation azimuths to constitute the observed values. For the predicted values, we manually measured the azimuths between monuments in QGIS and systematically adjusted them to the magnetic system used in 1939 and 1965, with a declination of 5.5°E, to enable comparison. We derived this declination from USGS topographic maps published in 1934, 1937, and 1939, upon which Dr. Happ annotated his transects in 1965^81–83^. Again, there is considerable agreement between predicted and observed values with an R^2^ of 0.9977 (Fig. 26b)—54% of the value pairs are within 1° of each other. Errors yielded from transects where one, or none, of its monuments were recovered do not significantly influence the RMSE of 5.63°—their omission only improves it by 0.29°. Linear shape fidelity in terms of orientation is also preserved to a high degree with an RMSE that is less sensitive to monument recovery status than that for length.

This analysis suggests that the only significant potential indicator of diminished shape fidelity is monument recovery status. Despite major variation in local environmental factors such as open-sky obstruction at a monument and in procedural factors such as the survey equipment–correction employed, the linear correlations exhibited in Fig. 26 indicate that their influence is minimal. For transects where both monuments were last recovered, we deem it appropriate to propagate GIS-layer-element positional accuracies from our previously reported GNSS-coordinate accuracies of 0.25–5 m. Where a monument remains unrecovered, this operation would yield minimum uncertainty values.

## Data Availability

The Python codes we developed to digitize and analyze the stream-and-valley-sedimentation-survey data for the Whitewater River Valley are available on GitHub and Zenodo^84^. Our repository contains four programs that (1) digitize the transect elevation data, (2) calculate mean-transect-elevation-change rates, (3) perform statistical ANOVA tests, and (4) house our user-defined functions. See the repository’s README for a brief explanation of their function and a user guide^84^.
